# Efficient and Precise Processing of the Optimized Primary Artificial MicroRNA in a Huntingtin-Lowering Adeno-Associated Viral Gene Therapy *In Vitro* and in Mice and Nonhuman Primates

**DOI:** 10.1089/hum.2021.221

**Published:** 2022-01-17

**Authors:** Wei Wang, Pengcheng Zhou, Xin Wang, Fen Chen, Emily Christensen, Jeffrey Thompson, Xiaoqin Ren, Adrian Kells, Lisa Stanek, Todd Carter, Jay Hou, Dinah W.Y. Sah

**Affiliations:** ^1^ Voyager Therapeutics, Cambridge, Massachusetts, USA; ^2^ Sanofi-Genzyme, Framingham, Massachusetts, USA.

**Keywords:** RNAi, artificial microRNA, Huntington's disease, huntingtin lowering, *in vivo*

## Abstract

Huntington's disease is a fatal neurodegenerative disorder caused by an inherited mutation in the huntingtin (*HTT*) gene comprising an expanded cytosine-adenine-guanine (CAG) trinucleotide repeat sequence that results in a pathogenic huntingtin protein. Adeno-associated viral (AAV) gene therapy containing a primary artificial microRNA (pri-amiRNA) specifically targeting *HTT* messenger RNA (mRNA) has the potential to provide long-lasting therapeutic benefit, through durable reduction of mutant *HTT* expression after a single administration. The efficiency and precision of processing of the pri-amiRNA precursor to the mature guide (G) strand by transduced cells are critical for specific and potent *HTT* mRNA lowering. The selection of the optimized pri-amiRNA comprised a series of *in vitro* studies followed by *in vivo* studies in small and then large mammals. Our studies demonstrate the predictivity of certain cell culture systems and rodent models for nonhuman primates with respect to some, but not all key features of pri-amiRNA processing. In addition, our results show that the processing of pri-amiRNAs to the mature guide strand can differ greatly across different scaffolds and sequences while providing the same levels of target lowering. Importantly, our data demonstrate that there is a combinatorial effect of guide and passenger (P) strand sequences, together with the scaffold, on pri-amiRNA processing, with different guide and passenger strand sequences within the same scaffold dramatically altering pri-amiRNA processing. Taken together, our results highlight the importance of optimizing not only target lowering but also the efficiency and precision of pri-amiRNA processing *in vitro*, in rodents and in large mammals to identify the most potent and selective AAV gene therapy that harnesses the endogenous microRNA (miRNA) biogenesis pathway for target lowering without perturbing the endogenous cellular miRNA profile. The optimized pri-amiRNA was selected with this focus on efficiency and precision of pri-amiRNA processing in addition to its pharmacological activity on *HTT* mRNA lowering and general tolerability *in vivo*.

## Introduction

Huntington's Disease (HD) is a rare, autosomal-dominant, debilitating, and fatal neurodegenerative disorder characterized by progressive motor, cognitive, and neuropsychiatric impairment. There are no effective therapies. Clinical onset based on motor diagnosis occurs at ∼40 years of age on average, with death typically 15–20 years later, preceded by profound physical deterioration and dementia.

HD is caused by a mutation in the huntingtin (*HTT*) gene, in which an expansion of more than ∼39 cytosine adenine guanine (CAG) trinucleotide triplet repeats results in a pathologic polyglutamine expansion in the N-terminus of the *HTT* protein. The resultant mutant huntingtin protein (mtHTT) has aberrant interactions with other proteins, leading to the disruption of cellular function and ultimately widespread neurodegeneration throughout the brain.^1^ The striatum and cortex are especially vulnerable to early neurodegeneration so protecting these regions is essential for the effective treatment of HD.

The *HTT* protein is a ubiquitous 3,144 amino acid protein that plays a role in multiple cellular processes. Numerous studies support the critical role of mtHTT in driving HD pathogenesis, likely through toxic gain of function of mtHTT and aberrant protein interactions.^2^ Several key neuronal functions are disrupted by mtHTT, including transcription, mitochondrial function, neurotransmitter vesicle recycling, and axonal transport. It is likely that no single aberrant effect of mtHTT is responsible for all aspects of neuronal dysfunction and subsequent cell death. Thus, reducing mtHTT expression is more likely to be of therapeutic benefit than targeting individual downstream consequences of mtHTT expression. To catalyze the degradation of mtHTT messenger RNA (mRNA), drugs that harness RNA interference (RNAi) can be developed that target mtHTT expression, potentially reducing or preventing all of these downstream effects.

While wild-type (WT) *HTT* protein has multiple cellular functions, it is anticipated that partial lowering of both WT *HTT* protein and mtHTT will be safe and effective in humans based on studies in transgenic mouse models^3–6^ and naive nonhuman primates (NHPs).^7,8^ Moreover, humans with a 50% reduction in WT *HTT* protein due to a mutation affecting one of the *HTT* alleles have a normal phenotype.^9^ Studies in transgenic rodent models of HD that recapitulate molecular, neuropathological, and behavioral aspects of HD have shown that partial simultaneous lowering of WT *HTT* and mtHTT mRNA with RNAi by up to ∼75% is well tolerated and results in the same phenotypic benefit as reducing mtHTT alone.

Although RNAi is durable for weeks, target lowering requires continuous exposure to the microRNA (miRNA). This necessitates repeat dosing of synthetic oligonucleotides, which is particularly challenging using direct routes of administration to the central nervous system (CNS). In contrast, the pharmacological effects of a one-time administration of adeno-associated viral (AAV) gene therapy are expected to remain clinically durable in the CNS. For example, in patients with Parkinson's disease who received AAV serotype 2 (AAV2) to deliver the enzyme aromatic amino acid decarboxylase to the putamen, expression was maintained for at least 4 years in the putamen.^10^ AAV-mediated RNAi gene therapy for the CNS would circumvent the need for multiple administrations which are required for treatment with synthetic oligonucleotides.

The vast majority of AAV gene therapies in development (∼200 active or completed trials listed on clinicaltrials.gov as of March 31, 2021) have comprised gene addition or replacement approaches, intended to increase the expression of genes that are transcribed at abnormally low levels or to replace genes that are missing entirely, respectively. Relatively few clinical studies to date have been conducted with AAV-mediated RNAi gene therapies despite their tremendous clinical potential to reduce the expression of genes that cause or contribute to disease. These trials comprise studies of AAV-mediated RNAi gene therapies to treat amyotrophic lateral sclerosis (ALS) with mutations in superoxide dismutase 1^11^ and HD.^12^ The considerations around clinical candidate selection and optimization for an inhibitory transgene comprising an RNAi payload differ substantially from those for a gene replacement payload. Although in both cases a target range of pharmacologic modulation needs to be achieved that provides therapeutic benefit and is safe, selectivity and tolerability parameters related to transgene expression are very different for the two approaches.

For an efficacious and safe AAV gene therapy delivering an RNAi payload, it is important to consider the mechanism of action with respect to harnessing the endogenous miRNA biogenesis pathway after cell transduction and transcription of the transgene.^13–16^ In the canonical miRNA biogenesis pathway, whether the artificial small interfering RNA (siRNA) is in a natural or artificial miRNA scaffold, the primary miRNA will form a hairpin-like secondary structure with flanking arms that will be cleaved by Drosha in the nucleus to the stem-loop precursor-miRNA (pre-miRNA) which is the short hairpin RNA (shRNA) structure used in some AAV gene therapies. This pre-miRNA is then exported from the nucleus to the cytoplasm by exportin 5, where the second cleavage by Dicer generates the mature miRNA duplex containing the mature guide (G) and passenger (P) strands. The mature guide strand is loaded into Argonaute to form a miRNA-induced silencing complex (miRISC)^17^ that selectively cleaves or suppresses the target mRNA after complementary base pair (bp) matching with the guide strand. In particular, the seed sequence (positions 2–8 counting from the 5′ end of the mature guide strand) is critical for initiating target mRNA recognition, binding, and degradation.^18,19^ Thus, accuracy of 5′-end processing of the guide strand is essential for maintaining fidelity of the seed sequence and target mRNA degradation, and consequently, shifts of the 5′ end should be avoided. The passenger strand, by contrast, is degraded rapidly and does not form miRISC. Nonetheless, a high guide-to-passenger (G/P) strand ratio is preferred to ensure that any effects of increased levels of the passenger strand are minimized.

Overharnessing the endogenous miRNA biogenesis pathway has been shown to lead to cytotoxicity in multiple organs in mice, rats, and dogs.^13–15,20–24^ This toxicity has been attributed to a perturbation of the balance of endogenous miRNAs required for multiple normal cellular processes and concomitant saturation of the nuclear RNAi export machinery with accumulation of pre-miRNA. The use of artificial miRNA scaffolds, rather than shRNAs,^23^ appears to mitigate this toxicity by increasing the efficiency of RNAi processing and reducing the levels of exogenous RNAi molecules while maintaining target lowering. Examples of this in the CNS include AAV-mediated RNAi targeting *HTT* mRNA for HD,^14–16,25,26^ ataxin-1 for spinocerebellar ataxia type 1,^27^ and ataxin-3 for spinocerebellar ataxia type 3.^28^ However, even with artificial miRNA scaffolds, there is a possibility that the endogenous miRNA biogenesis pathway is overharnessed. Therefore, monitoring the expression level of mature exogenous miRNA is essential.

Taken together, these considerations suggest that attributes of a potent, selective, and safe AAV gene therapy harnessing RNAi for target lowering should comprise efficient and precise processing of the primary artificial microRNA (pri-amiRNA) precursor to the mature guide strand in transduced cells, with (A) low levels of expression of the mature exogenous miRNA, (B) a high G/P strand ratio, and (C) high accuracy of 5′-end processing of the mature guide strand. To date, there have been five studies characterizing miRNA processing for AAV-mediated RNAi gene therapies. Most of these studies have focused on miRNA processing *in vitro*^29–32^ and in rodents^29,33^ with only one study in large mammals.^34^ Accuracy of 5′ end processing of the mature guide strand and G/P strand ratios were reported for both *in vitro* and *in vivo* systems, but the level of mature exogenous miRNA relative to the total endogenous pool of miRNAs was only characterized *in vitro*. Thus, there remains a key gap in our understanding of the *in vivo* performance of AAV-delivered pri-amiRNAs with respect to their impact on the endogenous miRNA biogenesis pathway.

In this study, we describe a series of *in vitro* studies in multiple cell types and *in vivo* studies in YAC128 mice and NHPs to compare *HTT* mRNA lowering and pri-amiRNA processing parameters for multiple pri-amiRNAs. Our results demonstrate the predictive power of *in vitro* and rodent models for NHPs with respect to some but not all key parameters of pri-amiRNA processing. Furthermore, our data highlight dramatic differences in processing of pri-amiRNAs to the mature exogenous miRNA across different scaffolds and miRNA sequences despite indistinguishable levels of target lowering. Moreover, our results show a combinatorial effect of guide and passenger strand sequences paired with different scaffolds on pri-amiRNA processing, underscoring the importance of simultaneous scaffold, guide, and passenger sequence selection to optimize pri-amiRNA processing. In summary, our results demonstrate that the efficiency and precision of pri-amiRNA processing are key characteristics to evaluate and optimize to identify a potent, selective, and safe AAV gene therapy that harnesses RNAi for target lowering. Our optimized pri-amiRNA was selected with this focus on efficiency and precision of pri-amiRNA processing, in addition to *HTT* mRNA lowering and general tolerability *in vivo*.

## Materials and Methods

### Constructs and vectors

AAV vectors (Supplementary Fig. S1) were produced by Voyager Therapeutics or the University of Massachusetts Chan Medical School Viral Vector Core. The transgene DNA comprised AAV2-derived inverted terminal repeats flanking a chicken β-actin (CBA) promoter, a pri-amiRNA scaffold containing a miRNA targeting human *HTT* mRNA, a rabbit beta-globin polyadenylation (pA) signal, and a stuffer sequence (iHtt). The positive control pri-amiRNA targeting *HTT* mRNA comprised our modifications of the previously published sequence mi2.4,^14^ with the modifications consisting of the removal of two adenine nucleotides at the 3′ end of the seed sequence in the passenger strand, creating a bulge due to the remaining thymine nucleotides in the guide strand, and the addition of an adenine nucleotide at the 3′ end of the passenger strand and a guanine nucleotide at the 3′ end of the guide strand.

The transgene DNA was packaged in a recombinant AAV1 or AAV2 capsid. Recombinant AAV vectors were produced either by triple transfection of adherent HEK293 cells or with the Sf9/baculovirus system and purified using previously described protocols.^35–39^ In brief, recombinant vectors were purified from clarified lysates using iodixanol gradients or by the cesium chloride gradient sedimentation method. Vectors were formulated in phosphate-buffered saline with 0.001% (w/v) Pluronic F-68^®^, pH 7.2–7.4, with or without 55–110 mM sodium chloride, and stored at less than or equal to −60°C. Vector titers were measured by droplet digital polymerase chain reaction (ddPCR) and/or quantitative polymerase chain reaction (qPCR). For vectors used for *in vivo* studies, the relative purity of the capsid proteins was confirmed using polyacrylamide gel electrophoresis (PAGE) followed by silver staining analysis. Genome integrity was evaluated by denaturing 1% agarose gel electrophoresis followed by SYBER Gold staining. In addition, the percentage of full capsids by cesium chloride gradient was at least 70%. Aggregation was <25%, and endotoxin levels were <2 endotoxin units (EU)/mL by the limulus amebocyte lysate test.

Vector formulation and aliquoting were generally performed 1 day before use. AAV vectors were removed from storage at less than or equal to −60°C and allowed to thaw at room temperature or on refrigerated cold packs, then maintained at ∼4°C. Dilutions were made with formulation buffer (192 mM sodium chloride, 10 mM sodium phosphate, 2 mM potassium phosphate, 2.7 mM potassium chloride, and 0.001% Pluronic F-68; pH 7.4) and then stored at ∼4°C until use. Vehicle groups received formulation buffer. For studies in NHPs, ProHance^®^ (gadoteridol; Cat. No. 111104; Bracco Diagnostics, Monroe Township, NJ) was added at a 1:250 dilution to all dosing solutions. Dosing solutions were brought to room temperature before intracranial infusion.

For *in vivo* comparison studies in both YAC128 mice and NHPs, dosing solutions were blinded and unblinded after the data had been analyzed.

### Cell culture and treatments

HeLa, U251 MG, and SH-SY5Y cells were cultured in Dulbecco's Modified Eagle's Medium (DMEM)/F12-GlutaMAX^™^ medium (Cat. No. 10565-018; Thermo Fisher Scientific, Waltham, MA) supplemented with 10% fetal bovine serum (FBS; Cat. No. 16000-044; Thermo Fisher Scientific), MEM Non-Essential Amino Acid Solution (Cat. No. 11140-050; Thermo Fisher Scientific), and HEPES.

For evaluation of endogenous *HTT* mRNA suppression, HeLa, U251MG, or SH-SY5Y cells were seeded in a 96-well plate at 1 × 10^4^, 2 × 10^4^, or 0.3 × 10^4^ cells/well, respectively, before transduction with Treatments A–P or AAV.mCherry (negative control) at a multiplicity of infection (MOI) of 1 × 10^5^ vector genomes (VG)/cell. HeLa cells were transduced with vector ∼24 h after plating. U251MG cells were mixed with vector just before plating. Before vector transduction, SH-SY5Y cells were neuronally differentiated. SH-SY5Y cells were plated on tissue culture plastic coated with 100 μg/mL Poly-d-Lysine (Cat. No. A-003-E; MilliporeSigma, St. Louis, MO) and 10 μg/mL laminin (Cat. No. 23017-015; Thermo Fisher Scientific) and cultured in differentiation medium comprising DMEM/F12-GlutaMAX medium supplemented with MEM Non-Essential Amino Acids Solution, sodium pyruvate, 0.5% FBS, 50 nM human recombinant IGF-1 (Cat. No. AF-100-11; Peprotech, Cranbury, NJ), and HEPES for 4 days before addition of vector. Each vector was tested in triplicate. At 48, 24, or 36 h post-transduction of HeLa, U251MG, or neuronally differentiated SH-SY5Y cells, respectively, the cells were harvested and lysed for analysis of endogenous *HTT* mRNA levels.

For the dual-Luc assay, HeLa cells were seeded in a 96-well plate at 8 × 10^3^ or 1 × 10^4^ cells/well, transfected with 1.25 ng/μL *HTT* pri-amiRNA plasmid in triplicate using FuGENE^®^ HD (Cat. No. E2311; Promega, Madison, WI), and lysed at 48 h post-transfection. For small RNA sequencing, HeLa and neuronally differentiated SH-SY5Y cells were cultured in 10 or 15 cm plates.

HEK293T cells were cultured in complete DMEM/F12-GlutaMAX medium supplemented with 10% FBS. For evaluation of endogenous *HTT* mRNA and protein suppression, the cells were seeded in a 96-well plate at 2 × 10^4^ cells/well 4 h before AAV.EGFP or transduction with Treatment P. The AAV vector was added to cultured HEK293T cells at a MOI of 3.0 × 10^2^ to 3.0 × 10^5^ VG/cell. Each vector dilution was tested in duplicate for *HTT* mRNA determination and on a single culture for *HTT* protein determination. After 24, 48, 72, or 96 h, the cells were harvested and lysed for analysis of *HTT* mRNA and protein levels. For the dual-Luc assay, HEK293T cells were seeded in a 96-well plate at 2.5 × 10^4^ cells/well, transfected with 1.25 ng/μL *HTT* pri-amiRNA plasmid in triplicate using FuGENE HD, and lysed at 48 h post-transfection.

HeLa S3 cells used for the AAV neutralizing antibody (NAb) assay were cultured in complete DMEM supplemented with 10% FBS, 100 units/mL penicillin, and 100 μg/mL streptomycin. One day before AAV transduction, the cells were seeded in a 96-well plate at 1 × 10^4^ cells/well. AAV1.eGFP was preincubated with the NHP serum sample at 37°C for 1 h and then added to cultured HeLa S3 cells at a MOI of 4 × 10^4^ VG/cell. Each condition was tested at dilutions of 1:16 and 1:64. After 48 h, the relative percentage of GFP positive cells was determined by flow cytometry.

### Anti-AAV1 NAb assay

To screen NHPs for low or no detectable levels of anti-AAV1 NAb activity in the serum before inclusion in the study, an *in vitro* anti-AAV1 NAb assay was used to measure the ability of NHP serum to inhibit AAV1.eGFP transduction of HeLa S3 cells. AAV1.eGFP was preincubated with the NHP serum sample at 37°C for 1 h and then added to cultured HeLa S3 cells at a MOI of 4.00 × 10^4^ VG/cell and a NHP serum dilution of 1:16 or 1:64. After 3 days, the cells were dissociated with 0.25% trypsin-EDTA to generate a single cell suspension for analysis on a flow cytometer (BD Accuri C6; Becton Dickinson, Franklin Lakes, NJ). Fixable Viability Stain-660 (Cat. No. 564405; Becton Dickinson) and then Cytofix^™^ solution (Cat. No. 554655; Becton Dickinson) were added to the cells, to analyze viable cells for GFP signal. The percentage of GFP-positive versus GFP-negative cells (FL-1 channel) was determined. The relative number of GFP-positive cells was then calculated by normalizing the percentage of GFP-positive cells with AAV1-eGFP treatment in the presence of NHP serum to the percentage of GFP-positive cells with AAV1-eGFP treatment in the absence of NHP serum. NHPs with sera that resulted in <50% inhibition of AAV1.eGFP transduction in HeLa S3 cells at 1:16 dilution (*i.e*., at least 50% GFP-positive cells) and <20% inhibition at 1:64 dilution (*i.e*., at least 80% GFP-positive cells) were defined as animals with no detectable or low levels of serum anti-AAV1 NAb activity and selected for inclusion in the study.

### Animals and treatments

#### YAC128 mice

All animal procedures were approved by the Institutional Animal Care and Use Committee (IACUC) at Sanofi-Genzyme (Department of Health and Human Services, NIH Publication 86–23). *Mus musculus* FVB/YAC Tg(+) mice (YAC128),^40^ 2–3 months of age, were placed on study and randomly assigned into treatment groups by even distribution of gender and age. The mice were maintained on a 12-h light/12-h dark cycle with food and water available *ad libitum*.

For administration of either vehicle or AAV1-iHtt, animals were anesthetized using 3% isoflurane and placed into a stereotaxic frame. All animals received bilateral injections into the striatum (anteroposterior, +0.50; mediolateral, ±2.2; dorsoventral, −3.0 from bregma and dura; incisor bar, 0.0) of 0.5 μL/min for 10 min using a stereotactically positioned 10 μL Hamilton syringe. The syringe was left in place for 1 min before withdrawal.

#### Nonhuman primate

The in-life portion of the study was performed at Valley Biosystems (West Sacramento, CA). All animals were housed in accordance with the Animal Welfare Act (9CFR Parts 1, 2, and 3) and The Guide for the Care and Use of Laboratory Animals (National Academy Press, Washington, DC, 2011). All animal procedures were approved by the IACUC and carried out under an IACUC protocol. Male and female rhesus macaques (*Macaca mulatta*), approximately 4–10 years of age and 5–11 kg body weight, were placed on study, after screening for serum levels of AAV1 NAb before study commencement. Twenty-one animals with serum AAV1 NAb titers <1:16 were randomly assigned to treatment groups, with six animals per AAV vector group and three animals in the vehicle group. The animals were maintained on a 12-h light/12-h dark cycle on a supplemented Harlan Primate diet, with water available *ad libitum*.

For administration of either AAV vector or vehicle, each animal was sedated with an intramuscular injection of ketamine (Ketaject; 10 mg/kg) and dexmedetomidine (0.015 mg/kg). Tracheal intubation was performed, and the animal was placed on inhaled isoflurane (1.5–4%) for the duration of the guide cannula implantation, magnetic resonance imaging (MRI) and dosing, and the guide cannula removal. The head was placed in a stereotaxic frame and after bilateral occipital craniotomies over the infusion target (putamen), two guide cannulae were temporarily implanted. Real-time MRI during infusion was used to guide the intracranial dosing procedure which comprised 100 μL per side at 3–5 μL/min. After intracranial dosing, removal of the guide cannulae, implant removal, and closure of surgical incisions, the animal was extubated and administered sedation reversal, postoperative analgesic, anti-inflammatory treatments, and antibiotics before being returned to its home cage. The animal was visually monitored until full recovery from anesthesia.

### General tolerability assessments

#### YAC128 mice

Cage side observations were conducted before injection and then performed daily after surgery. All mice were monitored for general health and signs of pain and/or illness daily. For body weights, mice were weighed immediately before injection and then once weekly after surgery. Brain weights were assessed at necropsy in the comparison study of 16 precandidate pri-amiRNAs for all mice except one animal in the Treatment G group whose brain weight was inadvertently not measured.

#### Nonhuman primate

Assessments included cage-side observations, body weights, food consumption, and clinical pathology, beginning 8 days before dosing. Cage-side observations were performed twice daily until euthanasia for general health, abnormalities, including neurological signs, and signs of pain or distress. Body weights were taken weekly, and food consumption was qualitatively assessed daily. Serum clinical chemistry, coagulation, and hematology were evaluated in blood samples collected ∼8 days before dosing and ∼15 and 36 (immediately before necropsy) days postdosing.

### Tissue collection

#### YAC128 mice

Mice received an intraperitoneal injection of phenytoin (Euthasol^®^; 270 mg/kg). Once unresponsive, mice were perfused transcardially with cold phosphate buffered saline, and the brains were removed. Brains were cut along the coronal axis using a mouse brain matrix (Harvard Apparatus, Holliston, MA) and striatal regions were dissected from a 2 mm brain slab using a 3 mm biopsy punch. Brain samples were then flash-frozen in liquid nitrogen and stored at −80°C.

#### Nonhuman primate

Animals were euthanized on days 36 ± 3 with intravenous pentobarbital sodium (100 mg/kg). Bilateral thoracotomy and transcardial perfusion with ice-cold saline were performed, followed by gross examination of major peripheral organs. Brains were removed and placed in a monkey brain matrix to collect coronal brain slabs of 3 mm thickness that were frozen and stored at −80°C. For collection of putamen samples, brain slices were warmed at room temperature for 1 min, and then 2 mm diameter punches were rapidly collected, transferred into a precooled collection tube on dry ice, and stored at −80°C until analysis.

To evaluate *HTT* mRNA levels in the putamen, 10 punches were collected from each putamen. Since there were 6 NHPs treated with each AAV1-iHtt vector and since each animal received 2.7 × 10^11^ VG in the left putamen and 9 × 10^10^ VG in the right putamen of the same AAV1-iHtt vector, a total of 60 punches was evaluated for each dose of each AAV1-iHtt vector (6 animals, 10 punches per dose, 120 punches per animal). For the vehicle group comprising 3 NHPs, 60 punches were evaluated (10 punches per putamen, 2 putamina per animal).

For small RNA deep sequencing, one punch was collected from each putamen, resulting in a total of six punches for each dose of each AAV1-iHtt vector and six punches from the vehicle group.

### Dual-Luc assay

The dual-Luc assay (Dual-Luciferase^®^ Reporter 1000 Assay System; Cat. No. E1980; Promega) was conducted in HEK293T and HeLa cells. Reporter plasmids for evaluating guide and passenger strand activity contained the target regions of the tested set of guide or passenger strands in tandem, separated by 2–6 nucleotides. *HTT* pri-amiRNA and reporter plasmids were transfected into the cells at 1.25 and 0.025 ng/μL, respectively, in triplicate using FuGENE HD, and then 48 h after transfection, the cells were lysed and the Renilla and firefly luciferase activities measured, according to the manufacturer's instructions. The relative activity was obtained by normalizing the Renilla luciferase level to the control firefly luciferase level.

### *HTT* mRNA levels

#### Human *HTT* mRNA levels *in vitro*

Levels of human *HTT* and X-prolyl aminopeptidase 1 (XPNPEP1) mRNA *in vitro* were measured by reverse transcription-quantitative polymerase chain reaction (RT-qPCR). Reverse transcription was performed with the TaqMan^™^ Gene Expression Cells-to-CT^™^ Kit (Cat. No. AM1729; Thermo Fisher Scientific) and TaqMan primer probe sets specific to human *HTT* (FAM labeled) and human XPNPEP1 (VIC labeled). qPCR was performed using the CFX384 real-time PCR system (Bio-Rad, Hercules, CA), and data were analyzed with the 2^−ΔΔCt^ method. Human *HTT* mRNA levels were normalized to human XPNPEP1 mRNA levels, and the normalized corresponding dose of AAV2.mCherry or AAV2.eGFP control group was used as a comparator.

#### Human *HTT* mRNA levels in YAC128 mice

Levels of human mutant *HTT* and mouse XPNPEP1 mRNA in YAC128 mouse striatum samples were measured by RT-qPCR using TaqMan primer probe sets specific for detecting human *HTT* and mouse XPNPEP1 mRNA. Striatal samples were partially thawed on ice and total RNA extracted using the RNeasy Mini Kit (Cat. No. 74104; Qiagen, Hilden, Germany) according to the manufacturer's protocol. Complementary DNA (cDNA) synthesis was performed by reverse transcription using the QuantiTect^®^ Reverse Transcription Kit (Cat. No. 205314; Qiagen). All TaqMan assays and master mixes (Thermo Fisher Scientific) were used according to the manufacturer's recommendations. qPCR was performed using the CFX384 real-time PCR system (Bio-Rad), and data were analyzed with the 2^−ΔΔCt^ method. Human *HTT* mRNA levels were normalized to mouse XPNPEP1 mRNA levels, and the normalized vehicle control group was used as a comparator.

Samples with cycle threshold (Ct) values equal to or greater than 35 were defined as negative and excluded from data analyses. cDNA samples were considered to have no genomic DNA contamination if the Ct value was equal to or greater than 35 for the corresponding no reverse transcription control RNA sample. The ROUT test (GraphPad Prism) was used for identification of outlier Ct values from the qPCR, and these outlier samples (Treatment F animal No. 28, Treatment G animal No. 94, and Treatment M animal No. 25) were excluded from further analysis.

#### *HTT* mRNA levels in NHP

Levels of NHP *HTT* mRNA in putamen samples were measured with a branched DNA (bDNA) assay using the QuantiGene^®^ 2.0 Plex Assay Kit (Cat. No. QP1015; Thermo Fisher Scientific) and specific probe sets for detecting rhesus *HTT* and three reference genes, TATA-box binding protein (TBP), alanyl-tRNA synthetase (AARS), and XPNPEP1 mRNA. Two-millimeter diameter punches from the putamen were weighed and then homogenized in QuantiGene Plex homogenization buffer with proteinase K (Cat. No. QS0106; Thermo Fisher Scientific) using the Geno/Grinder^®^ (Cat. No. 2010; SPEX Sample Prep, Metuchen, NJ). The resulting lysates were aliquoted and stored at −80°C until use in bDNA or VG DNA assays.

Immediately before use, the lysates were thawed and assayed according to the manufacturer's instructions. The plates were read on a MAGPIX instrument (Luminex, Austin, TX). Each lysate sample was assayed in duplicate.

*HTT* mRNA signals were normalized to the geometric mean of the signals of the three reference genes and then expressed relative to the vehicle group.

### *HTT* protein levels

Human *HTT* protein levels *in vitro* were measured by western blot. Cells were pelleted and lysed in MSD Tris Lysis Buffer (Cat. No. R60TX; Meso Scale Discovery, Rockville, MD) supplemented with protease and phosphatase inhibitor cocktail. Cell lysates were then cleared by centrifugation and supernatants evaluated for total protein by the BCA assay. A sample containing 20 μg protein was denatured at 70°C and run on a 3–8% Tris Acetate NuPAGE^™^ gel. Proteins were transferred onto PVDF membrane using the iBlot^™^ 2 transfer device (Invitrogen) according to the manufacturer's instructions. The PVDF membrane was treated with LI-COR Odyssey^®^ blocking buffer and then incubated with primary antibodies against β-actin (Cat. No. ab8227; Abcam, Cambridge, MA) or *HTT* protein (Cat. No. MAB2166; EMD Millipore, Danvers, MA), followed by secondary antibodies comprising goat anti-mouse IRDye 800CW conjugated secondary antibody (LI-COR; 800 nm, green) for *HTT* protein and rabbit goat anti-rabbit IRDye 680RD conjugated secondary antibody (LI-COR; 680 nm, red) for β-actin. After extensive washing, the PVDF membrane was scanned with the LI-COR Odyssey imaging system at 680 and 800 nm, and the fluorescence intensities for the *HTT* and β-actin protein bands were quantified using the LI-COR Odyssey imaging software. For each sample, the *HTT* protein signal was normalized to the corresponding β-actin signal.

### VG levels

The number of VG copies in putamen samples was measured with the ddPCR assay after DNA extraction. An aliquot of the frozen lysates prepared in QuantiGene Plex homogenization buffer with proteinase K as described above was thawed for whole cell DNA extraction using the DNeasy Blood & Tissue DNA Purification Kit (Cat. No. 69581; Qiagen, Germantown, MD). After combining the aliquot with an equal volume of ATL buffer, DNA was purified according to the manufacturer's instructions. The concentration of whole cell DNA was determined by NanoDrop (Thermo Fisher Scientific).

ddPCR was performed using a FAM labeled TaqMan probe set specific for the CBA promoter common to AAV1-iHtt-1, AAV1-iHtt-2, and AAV1-iHtt-3 and a VIC labeled probe set specific for the RNaseP gene of Rhesus macaque (Cat. No. 4403328; Thermo Fisher Scientific). These primer-probe sets (IDT, Coralville, IA), Supermix (Cat. No. 1863024; Bio-Rad), and HindIII-HF (Cat. No. R3104L; New England Biolabs, Ipswich, MA) were added to wells containing whole cell DNA. Droplets were generated using the Automatic Droplet Generator (Bio-Rad), and the reaction plate was then run on a thermocycler and analyzed using a ddPCR reader (QX-200; Bio-Rad). Values with ≤5 positive droplets per reaction were defined as below the limit of detection (LOD), based on no template controls which had no positive droplets or sporadic counts of 1–2 droplets per reaction, and excluded from analysis.

The number of VGs per diploid genome was calculated by dividing the copies/μL obtained with the CBA probe set by half the copies/μL obtained with the RNaseP probe set. The lower limit of quantification (LLOQ) of the assay was 0.01 VG copies/diploid genome.

### Small RNA sequencing and data analysis

#### RNA extraction and library preparation

For *in vitro* and YAC128 striatum samples, the RNA extraction and library preparation were performed at GENEWIZ (Cambridge, MA). Total RNA was isolated from *in vitro* or YAC128 striatum samples using the mirVana^™^ miRNA Isolation Kit, with Phenol (Cat. No. AM1560; Thermo Fisher Scientific). Striatum punch samples were weighed, and 10 × volume of lysis buffer (volume/weight) was added before homogenization in individual pestle/mortar tubes. After this, the manufacturer's protocol for RNA isolation was followed. Resulting RNA samples were quantified using the Qubit 2.0 Fluorometer (Life Technologies, Carlsbad, CA), and RNA integrity was measured with the Bioanalyzer 2100 (Agilent Technologies, Palo Alto, CA). A RNA integrity number (RIN) above 8.0 was required for sufficient quality of the RNA isolated from the sample to proceed to analysis of miRNA processing. All RIN numbers of RNA extracted from YAC128 mouse striatum samples were above 8 except for one sample from treatment N that had a RIN number of 7.8.

Small RNA sequencing libraries were prepared using Illumina TruSeq Small RNA Library Prep Kit (Cat. No. RS-200-0012; Illumina, San Diego, CA), starting from 1 μg of RNA according to the manufacturer's protocol. Amplified cDNA was purified by PAGE. The bands with correct sizes were excised from the gel, eluted with water, and concentrated by ethanol precipitation. The concentrations of the final libraries were assessed with the Qubit 2.0 Fluorometer and Bioanalyzer 2100. Libraries were pooled and sequenced on the Illumina HiSeq2500 with a read length of 50 bp. For NHP samples, small RNA library preparation methods were adapted from a protocol developed by the Zamore lab (UMass Medical Center).^41^ Total RNA was isolated at Voyager Therapeutics from each putamen punch using the mirVana miRNA Isolation Kit, with phenol (Cat. No. AM1560; Thermo Fisher Scientific). Tissue punch samples were weighed, lysed, and homogenized with a Geno/Grinder. RNA isolation was then performed according to the manufacturer's protocol. The resulting RNA samples were quantified using Nanodrop (Thermo Fisher Scientific), and RNA integrity was measured with the Bioanalyzer 2100 (Agilent Technologies). All RIN numbers of RNA extracted from NHP putamen samples were above 8.

Small RNA sequencing libraries were then prepared from the total RNA. Total RNAs were ligated to the 23 nucleotide (nt) 3′ adaptor AppBA3 (5′rAppTGGAATTCTCGGGTGCCAAGG/ddC/3′) using T4 Rnl2tr K227Q (Cat. No. M0351; New England Biolabs). Small RNAs ligated to the AppBA3 adaptor were separated from unligated adaptors using PAGE. The ligated products were recovered from minced gel pieces and then precipitated with ethanol.

The resulting small RNAs ligated to the 3′ adaptor were then ligated to the 26 nt 5′ RNA adaptor BA5 (5′GUUCAGAGUUCUACAGUCCGACGAUC3′). The concentrations of the final libraries were assessed using qPCR with an Illumina library quantification kit Kapa Biosystems (Cat. No. KK4844; Roche Sequencing and Life Science, Wilmington, MA). The libraries were pooled and sequenced on the Illumina NextSeq500 with a read length of 75 bp.

#### Next generation sequencing data analysis

The raw sequencing data were processed using an in-house pipeline. Raw small RNA sequencing data were 3′ adapter trimmed since the read length of 75 bp covered the small RNA (18–30 nt) and the prefix of 3′ adapter sequence. The 3′ adapter reference sequence was: 5′TGGAATTCTCGGGTGCCAAGG3′.

Reads filtered through the adapter trimming step were aligned to *Homo sapiens/M. musculus/M. mulatta* endogenous miRNA (miRbase v21)^42^ using the short-read aligning tool bowtie.^43^ Reads that failed to align to endogenous miRNA hairpins were aligned to the miRNA hairpin targeting *HTT* (mRNA miHTT) in the administered AAV vector using bowtie mapping, with the same parameters set to require a perfect match. The position of the mature miHTT relative to the miHTT hairpin was calculated. Coordinates of the detected miHTT mappers were then calibrated to that of the expected mature miHTT with an intersection cutoff rate of 0.75. These assessments allowed the percentage of miHTT guide strands containing precise 5′ ends to be calculated.

Guide strand mappers and passenger strand mappers were separated according to the pri-amiRNA design, and then the G/P strand ratio was calculated. When the overall expression of miHTT was relatively low, as for example with two NHPs administered AAV1-iHtt-1 at the low dose and one animal administered AAV1-iHtt-2 at the high dose, and the levels of passenger strand were below the LOD, the G/P strand ratio was excluded from the mean for the corresponding group.

The level of mature exogenous miHTT was normalized to the level of total endogenous miRNA and reported as reads per million or as percentage of endogenous miRNAs.

For each sample, the percentage of bases called by a sequencer with a Q Score of at least Q30 was required to be above 85% according to Illumina kit specifications. Reads without 3′ adapters or with low quality (less than Q20) were removed from further analysis. Only 3′ adapter trimmed reads perfectly aligned to reference hairpins were included for analysis.

## Results

**Figure 1.**
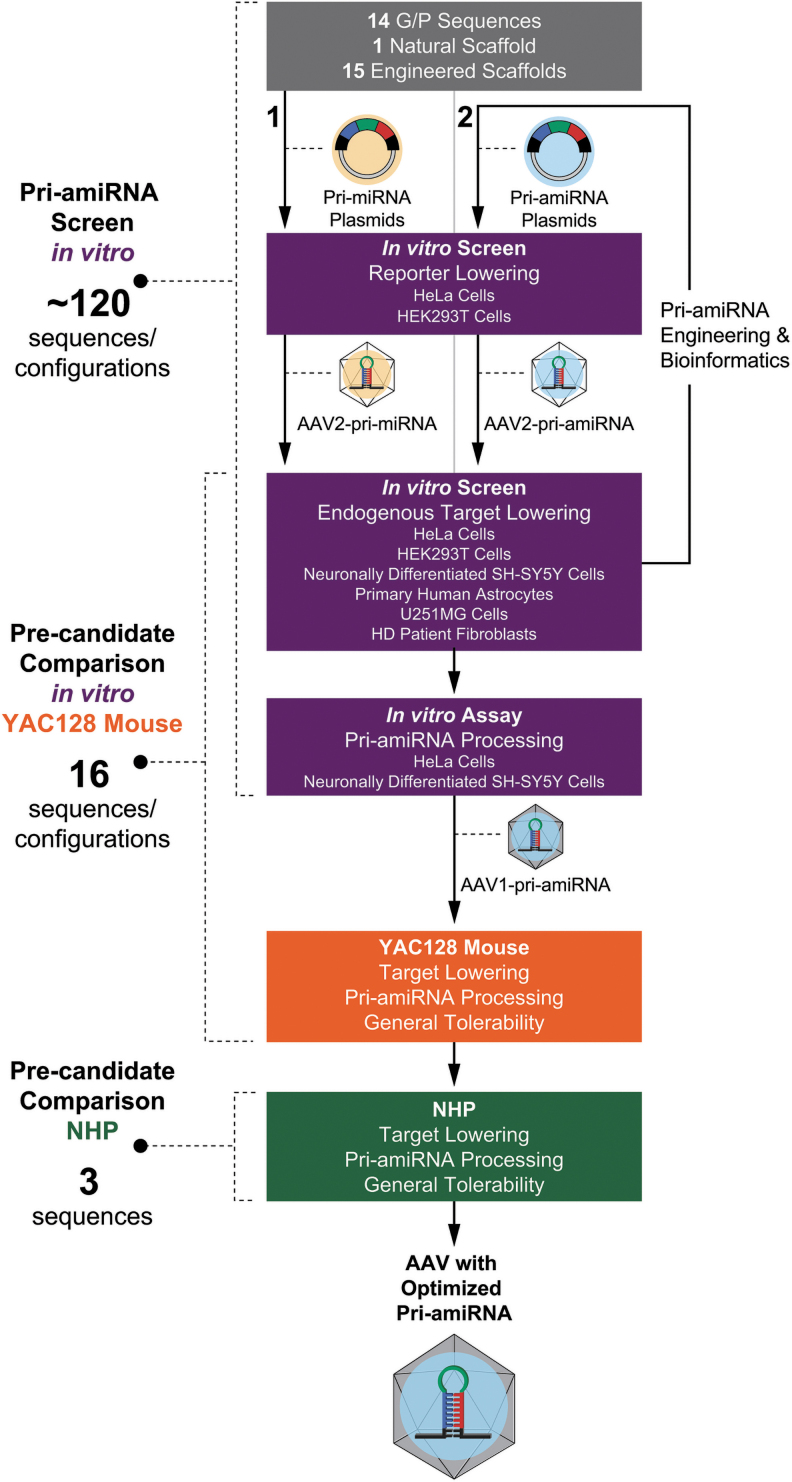
Diagram of pri-amiRNA optimization process and selection of the optimized pri-amiRNA. Initially **(1)**, guide and complementary passenger sequences were inserted into naturally occurring scaffolds and then evaluated *in vitro* in reporter assays and for endogenous target lowering. Through an iterative process **(2)** of engineering the scaffold and passenger strand and testing, pri-amiRNAs were identified that provided more effective target lowering *in vitro*. Sixteen pri-amiRNAs were selected for in-depth evaluation and designated precandidate pri-amiRNAs. This detailed characterization and comparison comprised target lowering and pri-amiRNA processing *in vitro* and in YAC128 mouse and NHP, as well as general tolerability *in vivo*. From these studies, the optimized pri-amiRNA was selected. AAV, adeno-associated viral; AAV1, AAV serotype 1; AAV2, AAV serotype 2; G, guide; NHP, nonhuman primate; P, passenger; pri-amiRNA, primary artificial microRNA. Color images are available online.

To select an optimized pri-amiRNA payload for an AAV gene therapy targeting *HTT* mRNA with RNAi for the treatment of HD, we engineered the artificial pri-microRNA, including the scaffold (stem, loop, and flanking arms), guide strand targeting *HTT* mRNA, and passenger strand in an extensive series of studies (Fig. 1). The ubiquitous CBA promoter was chosen to lower *HTT* mRNA across different cell types in the brain. Approximately 120 pri-amiRNA sequences and AAV VG configurations were screened *in vitro* in 6 different human cell types (HeLa cells, HEK293 cells, neuronally differentiated SH-SY5Y cells, primary human astrocytes, U251MG cells, and HD patient fibroblasts) for *HTT* mRNA lowering. The pri-amiRNA sequences initially included 14 different guide and companion passenger strand sequences selected based on *HTT* mRNA lowering and bioinformatic analysis, 1 naturally occurring pri-miRNA scaffold based on human brain expression, and 15 different engineered scaffolds derived from a miRNA expressed in human brain.

The primary goal of the modifications of the pri-amiRNA designs was to increase the processing efficiency of the pri-amiRNA by Drosha and Dicer for assembly of the guide strand into the RISC complex. These modifications were based, in part, on rules proposed from earlier studies.^44–51^ For example, the binding energy between the guide and passenger strands was optimized for strand separation. In addition, the sequence of the 5′ end of the mature miRNA guide strand was adjusted to maximize 5′ end processing accuracy to preserve the correct seed sequence. We then engineered and selected the scaffold, guide strand targeting *HTT* mRNA, and passenger strand in successive iterations based on bioinformatic analysis for potential passenger strand off-target effects, extent of *HTT* mRNA lowering *in vitro*, and passenger strand activity on reporter gene expression *in vitro*. These studies led to our detailed characterization and comparison of 16 precandidate pri-amiRNAs (Supplementary Fig. S1) *in vitro* and in YAC128 mice with respect to *HTT* mRNA lowering and pri-amiRNA processing, using a positive control (Supplementary Fig. S1), comprising the previously published sequence mi2.4^14^ with minor modifications, as a comparator. We then selected the top 3 precandidate pri-amiRNAs from these *in vitro* and YAC128 mouse studies to take forward into NHPs for selection of the optimized pri-amiRNA candidate. The candidate pri-amiRNA was chosen based on target lowering in the NHP putamen, efficiency and precision of pri-amiRNA processing, and general tolerability.

The *HTT* mRNA lowering and pri-amiRNA processing data from the 16 precandidate pri-amiRNAs, as well as considerations and process for selecting the optimal pri-amiRNA based on these data, are described below. These data reveal important insights into the relative importance of these *in vitro* and *in vivo* systems and highlight key parameters for optimizing a pri-amiRNA for an AAV gene therapy.

### Precandidate pri-amiRNA designs lower reporter and endogenous *HTT* mRNA levels efficiently *in vitro*

To identify pri-amiRNA candidates worthy of *in vivo* assessment, we first measured the *in vitro* activities of 16 precandidate pri-amiRNAs in human cell lines using a reporter assay designed to separately detect guide- and passenger-strand activity.

We conducted guide and passenger strand reporter assays in both HeLa and HEK293T cells following transfection of the precandidate pri-amiRNAs. From the starting pool of pri-amiRNA sequences, 16 precandidate pri-amiRNAs, Treatments A–P, were selected, primarily based on the guide strand reporter assay in which they were among the most effective pri-amiRNAs out of the starting pool in lowering the luciferase signal, and lowered the luciferase signal by more than 70% on average (Supplementary Fig. S2). In addition, these 16 precandidate pri-amiRNAs exhibited less than ∼30% lowering on average of the luciferase signal in the passenger strand reporter assay (data not shown). These findings demonstrate that the guide strands of these 16 precandidate pri-amiRNAs lowered their targets substantially, whereas the passenger strands of these precandidate pri-amiRNAs did not. Notably, all 16 precandidate pri-amiRNAs reduced the guide strand reporter signal similarly under these conditions.

Next, we evaluated the 16 precandidate pri-amiRNAs, Treatments A–P, for lowering of endogenous *HTT* expression. To determine useful *in vitro* conditions for comparing target lowering, we characterized the time course (Supplementary Fig. S3A) and dose dependence (Supplementary Fig. S3B, C) of *HTT* mRNA and protein lowering in HEK293T cells using Treatment P. *HTT* mRNA lowering demonstrated maximum reduction at approximately 24–48 h post-transduction with slight recovery of *HTT* mRNA levels at 72 and 96 h postdosing. *HTT* protein lowering slightly lagged *HTT* mRNA lowering with initial, partial *HTT* protein lowering at 24 h, and maximal *HTT* protein lowering attained at approximately 48–72 h post-transduction. At the 48-h timepoint post-transduction, which provided approximately maximum *HTT* mRNA and protein lowering, *HTT* mRNA and protein levels relative to vehicle showed similar dose–responses across MOIs of 3.0 × 10^2^ to 3.0 × 10^5^ VG/cell. Graded levels of *HTT* mRNA and protein lowering were observed at MOIs of 3.0 × 10^2^ to 9.5 × 10^3^ VG/cell with maximum levels of *HTT* mRNA and protein lowering attained at MOIs of 3.0 × 10^4^ to 3.0 × 10^5^ VG/cell. These results confirm that an MOI of 1.0 × 10^5^ VG/cell and a timepoint of 24–48 h post-transduction comprise optimal conditions for assessing *HTT* mRNA reduction *in vitro*.

**Figure 2.**
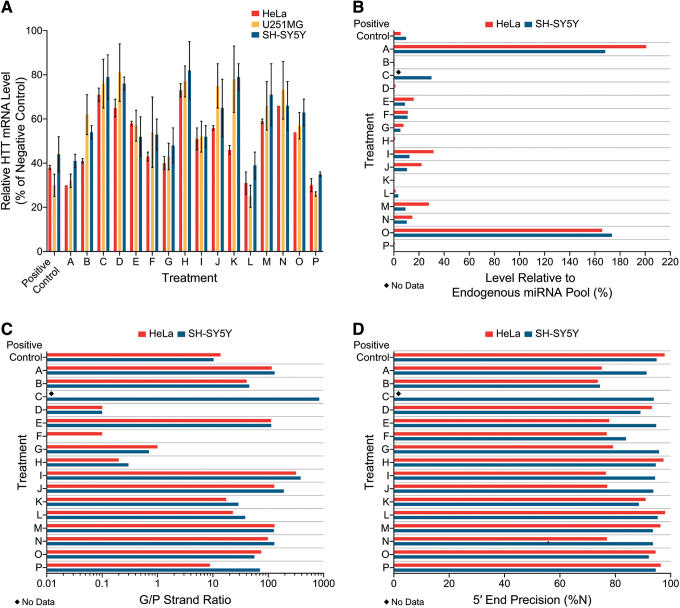
Human *HTT* mRNA lowering and pri-amiRNA processing *in vitro*. HeLa, U251MG, and neuronally differentiated SH-SY5Y cells were transduced with AAV2-packaged pri-amiRNAs targeting *HTT* mRNA at a MOI of 1.0 × 10^5^ VG per cell and harvested approximately 24–48 h later. Sixteen precandidate pri-amiRNAs (A–P) and a positive control were evaluated. **(A)** Human *HTT* mRNA levels, as well as mRNA levels of human XPNPEP1 as the endogenous reference gene, were measured by RT-qPCR. Human *HTT* mRNA levels were normalized to XPNPEP1 mRNA levels and then further normalized to the negative control group (AAV2.mCherry). All treatments were tested in triplicate and then samples were run in duplicate for qPCR. The group mean ± standard deviation is shown for each treatment. **(B–D)** Pri-amiRNA processing was evaluated by small RNA sequencing. *N* = 1 per treatment. **(B)** Level of mature exogenous miHTT relative to the total endogenous pool of miRNAs. **(C)** Ratio of guide to passenger strands of the mature exogenous miHTT. **(D)** Percentage of mature exogenous miHTT guide strands with precise 5′ ends. *HTT*, huntingtin; miRNA, microRNA; MOI, multiplicity of infection; mRNA, messenger RNA; RT-qPCR, reverse transcription-quantitative polymerase chain reaction; VG, vector genome; XPNPEP1, X-prolyl aminopeptidase 1. Color images are available online.

To evaluate the precandidate pri-amiRNAs for lowering of endogenous *HTT* mRNA, three different human cell lines were used. Since the primary target cell types for a *HTT* mRNA lowering therapy are neurons and glia, neuronally-differentiated SH-SY5Y cells, derived from a human neuroblastoma, and U251MG cells, derived from a human glioblastoma, were selected. In addition, *in vitro* testing was conducted in HeLa cells, which have been used widely to evaluate *HTT* mRNA lowering. Substantial differences between the 16 pri-amiRNAs emerged in these 3 cell lines when lowering of endogenous *HTT* mRNA was assessed (Fig. 2). For these studies, the 16 precandidate pri-amiRNAs were packaged in AAV2, which is more effective in transducing cells *in vitro* than AAV1 (used later for *in vivo* studies). All vectors were identical in construction, production, and purification, the only difference between the 16 vectors being the pri-amiRNA sequence. At an MOI of 1 × 10^5^ VG/cell, *HTT* mRNA lowering ranged from 27% to 70% in HeLa, 19% to 75% in U251MG, and 18% to 65% in neuronally differentiated SH-SY5Y cells, relative to the AAV2.mCherry control.

Pri-amiRNAs that lower the target mRNA to the greatest extent across multiple cell lines are more likely to be most effective in subsequent screens. The rank order of the 16 different precandidate pri-amiRNAs for lowering endogenous *HTT* mRNA was similar but not identical across the three cell lines (Fig. 2A). In HeLa cells, the rank order (most to least lowering of *HTT* mRNA on average) of precandidate pri-amiRNAs was: Treatment A, P > L > G > B > F > K > I > O > J > E > M > D > N > C > H. In U251MG cells, the rank order for *HTT* mRNA lowering was: Treatment L > P > A > G > I > F > E, O > B > M > N > J > C > H > K > D. In neuronally differentiated SH-SY5Y cells, the rank order for *HTT* mRNA lowering was: Treatment P > L > A > G > E, I > F > B > O > J > N > M > D > C, K > H. Thus, treatment with precandidate pri-amiRNAs P, L, A, and G resulted in the most *HTT* mRNA lowering on average in all three cell lines, with 59–75% lowering by precandidates L, P, and A and 52–60% lowering by precandidate G. The next (fifth) best precandidate pri-amiRNA in HeLa cells, Treatment B, resulted in more modest lowering of *HTT* mRNA in U251MG and neuronally differentiated SH-SY5Y cells than in HeLa cells. The fifth best precandidate pri-amiRNA in U251MG and neuronally differentiated SH-SY5Y cells, Treatment I was only ranked 8th in HeLa cells. The sixth best precandidate was Treatment F in all three cell lines. Interestingly, Treatment K performed well in HeLa cells, ranking seventh and producing 54% *HTT* mRNA lowering but was relatively ineffective at lowering *HTT* mRNA in U251MG and neuronally differentiated SH-SY5Y cells (by 22% or 21%, respectively), ranking 15th (second worst) in these two cell lines. These results show that the rank order of pri-amiRNAs in lowering endogenous target mRNA can vary substantially across different cell lines *in vitro*. Thus, *HTT* mRNA lowering in a single cell line may not be representative for selecting the most effective pri-amiRNA. These results demonstrate the importance of using multiple cell lines *in vitro* when assessing pri-amiRNA candidates.

### Extent of harnessing of cellular miRNA biogenesis pathway and guide/passenger strand ratio provide dramatic differentiation of precandidate pri-amiRNAs *in vitro*

We used high-throughput small RNA deep sequencing to evaluate the efficiency and precision of pri-amiRNA processing of the 16 precandidate pri-amiRNAs in HeLa and neuronally differentiated SH-SY5Y cells. These characteristics were compared to the reduction of *HTT* mRNA in these same cell lines (See Precandidate pri-amiRNA designs lower reporter and endogenous *HTT* mRNA levels efficiently *in vitro* section), using the same AAV vectors and MOIs.

Since minimal harnessing of the endogenous RNAi pathway is less likely to perturb the endogenous pool of miRNAs and more likely to leave their associated cellular functions intact, potent target lowering concomitant with low harnessing of the endogenous RNAi pathway are key characteristics for safety and efficacy considerations. In this study we evaluated the level of mature exogenous miHTT relative to the total endogenous pool of miRNAs in HeLa and neuronally differentiated SH-SY5Y cells (Fig. 2B) to monitor our precandidate's usage of the endogenous miRNA biogenesis pathway. The samples from HeLa and neuronally differentiated SH-SY5Y cells each had 13.7–33.8 million raw reads with the correct adapter sequence. First, we aligned the reads to annotated human miRNAs to identify the reads corresponding to endogenous miRNAs. We then aligned the remaining reads to our exogenous miHTT sequences. The level of mature exogenous miHTT relative to the total endogenous pool of miRNAs in HeLa and neuronally differentiated SH-SY5Y cells was then calculated. The abundance of mature exogenous miHTTs relative to the total endogenous pool of human miRNAs varied greatly across the 16 precandidate pri-amiRNAs, spanning a 1004-fold range from 0.2% for Treatment K in neuronally differentiated SH-SY5Y cells to 200.8% for Treatment A in HeLa cells. The expression levels of mature exogenous miHTTs from Treatments A and O exceeded the sum of all endogenous miRNA levels in the same sample (>100% of the endogenous miRNA pool), suggesting that these mature exogenous miHTTs out-competed endogenous miRNAs for loading into Argonaute. Although similar levels of *HTT* mRNA lowering, 59–65% on average, were achieved by precandidates A, L, and P in neuronally differentiated SH-SY5Y cells, the level of mature exogenous miHTT relative to the total endogenous pool of miRNAs varied from 0.6% for Treatment P to 168.2% for Treatment A, with Treatment L intermediate at 3.6%. Our data demonstrate that potent target lowering concomitant with low harnessing of the endogenous RNAi pathway is a key characteristic that provides strong differentiation *in vitro* between different pri-amiRNAs.

Since the guide strand is the strand intended to mediate *HTT* mRNA lowering using RNAi, while the passenger strand has no intended activity, higher G/P strand ratios are more favorable for potency and selectivity. Moreover, in the event that the passenger strand has unintended off-target activity, lower levels of the P strand would result in less off-target activity and, presumably, enhanced safety.

The G/P strand ratio varied substantially across the 16 precandidate pri-amiRNAs, from 0.1 to 317.6 in HeLa cells, and 0 to 834.4 in neuronally differentiated SH-SY5Y cells (Fig. 2C). Twelve of the 16 precandidate pri-amiRNAs exhibited G/P strand ratios >10 in neuronally differentiated SH-SY5Y cells (Treatments A–C, E, and I–P), as well as the positive control. In HeLa cells, transduction with 9 precandidate pri-amiRNAs packaged in AAV2 resulted in G/P strand ratios >10 (Treatments A, B, E, I–K, M–O). Treatment C was not evaluated for miHTT processing in HeLa cells, and Treatments L and P, as well as the positive control, were transfected using plasmids into HeLa cells so the results cannot be compared directly to those obtained with AAV transduction. Treatments D, F, G, and H showed G/P strand ratios <10 (range 0–1.0), including three precandidate pri-amiRNAs (Treatments D, F, and H) with ratios of <0.5, indicating that there were more than twice as many P strands as G strands. The G/P strand ratios were remarkably similar in HeLa and SH-SY5Y cells for a given pri-amiRNA, and the rank order for the precandidate pri-amiRNAs was likewise very similar in these two cell lines. Thus, the G/P strand ratio is a parameter that provides strong differentiation of different pri-amiRNAs and appears to be similar for a given pri-amiRNA across different cell lines *in vitro*.

Since the seed region is critical for recognition of the target mRNA and begins at a fixed position relative to the 5′ end of the guide strand, accurate processing of the 5′ end of the guide strand is required for the guide strand to bind with its intended target. Any shift in the 5′ end of the guide strand would result in an altered seed sequence that could interact with unintended mRNAs and potentially mediate off target effects. We assessed the precision of 5′ end processing of miRNA guide strands in HeLa and neuronally differentiated SH-SY5Y cells (Fig. 2D). In HeLa cells, seven precandidate pri-amiRNAs showed at least 90% correct 5′ end processing of the guide strand, whereas eight pri-amiRNAs exhibited <80% correct 5′ ends. Notably, four of these latter eight pri-amiRNAs were from the same scaffold, suggesting that the secondary structure of this scaffold may not favor accurate Dicer cleavage in HeLa cells. In neuronally differentiated SH-SY5Y cells, only one precandidate pri-amiRNA (Treatment B) showed <80% of guide strands with correct 5′ ends. All of the other 15 precandidate pri-amiRNAs were processed with at least 80% of guide strands with correct 5′ ends. These results indicate that all of our pri-amiRNA designs generated the desired seed sequences with good precision, but some pri-amiRNAs clearly outperformed others.

### Precandidate AAV1-iHtt vectors are well tolerated in YAC128 mouse

For *in vivo* screening of precandidate pri-amiRNAs, we selected the YAC128 mouse because it is a model of HD that would allow us to readily evaluate a substantial number of pri-amiRNAs for lowering of human *HTT* expression and pri-amiRNA processing, with the potential for future correlation of lowering of human *HTT* expression with phenotypic improvement. The YAC128 mouse is a yeast artificial chromosome animal model of HD with the entire human *HTT* gene containing 128 CAG repeats.^40^ This mouse model of HD is well characterized, has been used previously for evaluating *HTT* mRNA and protein lowering, and exhibits age-dependent striatal neurodegeneration and progressive motor and cognitive deficits.^40,52,53^ We compared the 16 precandidate pri-amiRNAs in the YAC128 mouse, with bilateral intrastriatal injection at total doses of 2.65 × 10^10^ to 6.00 × 10^10^ VG per animal, which were the maximum possible doses given the stock concentrations of vector. For *in vivo* studies, the pri-amiRNAs were packaged in AAV1, which distributes more broadly in the brain than AAV2 (used *in vitro*). Due to the large number of animals in the experiment, the study was divided into two cohorts, with each cohort having its own vehicle group.

All 16 precandidate AAV1-iHtt vectors (7–8 animals per AAV treatment) were well tolerated over the 4 weeks in-life period postdosing. Cage-side observations showed no abnormalities for all animals, and there were no significant differences in body weights, body weight changes, or brain weights measured at necropsy across groups (data not shown). An extensive tolerability evaluation is ongoing in the YAC128 mouse with much longer in-life periods postdosing that includes detailed histopathological and immunohistochemical examination, as well as efficacy in this model of HD; these results will be reported elsewhere.

### Precandidate pri-amiRNA designs lower *HTT* mRNA efficiently in YAC128 mouse striatum

**Figure 3.**
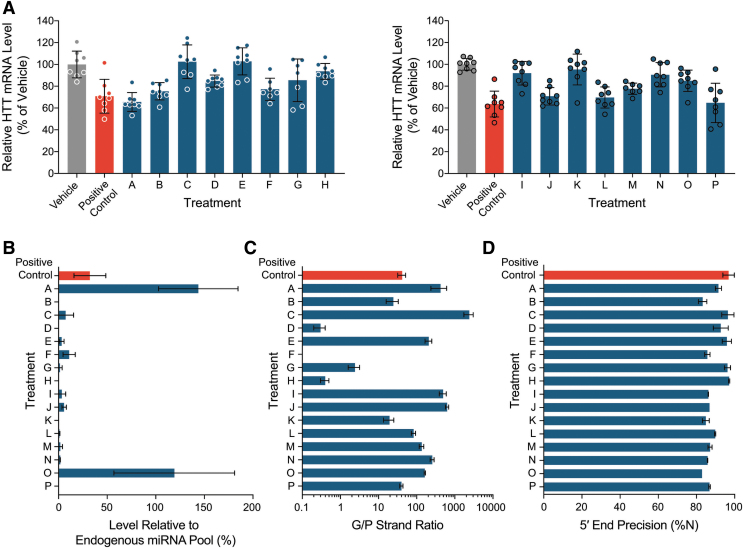
Human mutant *HTT* mRNA lowering and pri-amiRNA processing in YAC128 mouse striatum 4 weeks after bilateral intrastriatal injection of AAV1-packaged pri-amiRNAs targeting *HTT* mRNA at a dose of 2.6 × 10^10^ to 6.0 × 10^10^ VG per animal. Sixteen precandidate pri-amiRNAs (A–P), as well as a positive control and vehicle, were administered across two mouse cohorts (cohort 1: A–H; cohort 2: I–P), **(A)** Human mutant *HTT* mRNA levels, as well as mRNA levels of mouse XPNPEP1 and Hypoxanthine Guanine Phosphoribosyltransferase (HPRT) (endogenous reference genes), were measured by RT-qPCR. Human *HTT* mRNA levels were normalized to the geometric mean of XPNPEP1 and HPRT mRNA levels and then further normalized to the vehicle group of the cohort. Each symbol represents the average relative *HTT* mRNA level in the striatum of one animal. *N* = 8 for all groups except F, G, and M (*N* = 7). **(B–D)** Pri-amiRNA processing was evaluated by small RNA sequencing. *N* = 3 per group. The positive control samples are from cohort 1. **(B)** Level of mature exogenous miHTT relative to the total endogenous pool of miRNAs. **(C)** Ratio of guide to passenger strands of the mature exogenous miHTT. **(D)** Percentage of mature exogenous miHTT guide strands with precise 5′ ends. For all *panels*, the group mean ± standard deviation is shown for each treatment. Color images are available online.

We first evaluated *HTT* mRNA reduction in striatum samples from these YAC128 mice. Treatment with the 16 precandidate AAV1-iHtt vectors resulted in different levels of human mutant *HTT* mRNA reduction relative to their corresponding vehicle group at 4 weeks postdosing (Fig. 3A). Treatments A, B, F, J, L, M, and P, as well as the positive control, suppressed striatal human mutant *HTT* mRNA levels to a statistically significant extent (*p* < 0.05 by one-way analysis of variance [ANOVA] with Dunnett's multiple comparisons *post hoc* test), ranging from 22% to 35% lowering on average relative to the corresponding vehicle group. However, no significant human mutant *HTT* mRNA reduction was seen for treatments C–E, G–I, K, N, and O.

These results illustrate that greater discrimination across pri-amiRNAs is provided by comparison of human *HTT* mRNA reduction in YAC128 mice than *in vitro*, even with three different cell lines evaluated, including neuronal and glial cell types. The 16 precandidate pri-amiRNAs lowered human *HTT* mRNA *in vitro* by approximately 20–80% but only 7 significantly reduced human *HTT* expression *in vivo*. The most effective pri-amiRNAs *in vitro* that lowered human *HTT* mRNA by at least 55% in all three cell lines at an MOI of 1 × 10^5^ (*i.e*., Treatments A, L, and P and positive control) also lowered human *HTT* mRNA significantly in YAC128 mouse striatum. In addition, other pri-amiRNAs that were less effective *in vitro* (*i.e*., Treatments J and M) with as little as 25% human *HTT* mRNA lowering showed significant human *HTT* mRNA reduction *in vivo*. Conversely, there are examples of pri-amiRNAs that were substantially active *in vitro* with as much as 50% human *HTT* mRNA lowering (*i.e*., Treatment I) but not active *in vivo*. Notably, pri-amiRNAs with similar pharmacological activity *in vitro* such as Treatments F and I showed different pharmacological activity *in vivo*, indicating that target lowering *in vitro* may not completely predict target lowering efficiency *in vivo* for pri-amiRNAs packaged in AAV. These results highlight the importance of conducting an *in vivo* screen to identify the most effective pri-amiRNAs for target lowering. Nonetheless, given the much larger screening capacity *in vitro* than *in vivo* and the predictive power of certain pri-amiRNA processing parameters *in vitro* for *in vivo* results (See Pri-amiRNA processing efficiency and precision in YAC128 mouse striatum is similar to *in vitro* pri-amiRNA processing section), *in vitro* screens are useful for eliminating some precandidate pri-amiRNAs from further consideration.

### Extent of harnessing of cellular miRNA biogenesis pathway and guide/passenger strand ratio provide dramatic differentiation of precandidate pri-amiRNAs in YAC128 mouse striatum

Next, we evaluated the efficiency and precision of pri-amiRNA processing in striatum samples from 3 YAC128 mice per group (1 positive control, 16 precandidate pri-amiRNAs, and 2 vehicle groups), using small RNA deep sequencing. The data comprised 15.5–46.5 million raw reads per sample with the correct adapter sequence.

To evaluate endogenous miRNA profiles in these samples, the percentage of reads aligned to annotated *M. musculus* miRNA hairpin sequences was assessed (Supplementary Fig. S4). In the vehicle groups for cohorts 1 and 2, an average of 67.7% ± 4.4% and 57.0% ± 0.2% (*N* = 3, mean ± standard deviation) of reads, respectively, was aligned to annotated *M. musculus* miRNA hairpin sequences and thus represented endogenous miRNAs; these served as the baseline endogenous miRNA profile for the two cohorts in this study. In animals treated with Treatments B–H (cohort 1) or Treatments I–N and P (cohort 2), 60.9–68.3% and 50.8–59.8% of reads on average, respectively, were aligned to endogenous *M. musculus* miRNA hairpin sequences, with no significant difference from the baseline from the corresponding vehicle group (*p* > 0.05 by one-way ANOVA with Dunnett's multiple comparison *post hoc* test). However, the endogenous miRNA levels after administration of Treatment A (cohort 1), Treatment O (cohort 2), and the positive control (cohort 1), 29.2% ± 7.3%, 30.0% ± 8.2%, and 54.6% ± 1.8%, respectively, were significantly lower than the baseline in the corresponding vehicle group (*p* < 0.0001, *p* < 0.0001, and *p* = 0.03, respectively, by one-way ANOVA with Dunnett's multiple comparison *post hoc* test). These results suggest that the endogenous miRNA profiles in the striatum were significantly affected by administration of Treatments A and O, as well as the positive control, but not Treatments B–N and P.

The level of precandidate miRNA relative to the total endogenous pool of miRNAs was evaluated (Fig. 3B). For the 6 samples from vehicle-treated animals, the detectable miHTT read number (with all 16 precandidate pri-amiRNAs used as reference) was <85 rpm, resulting in expression levels of 0.0% ± 0.0% (mean ± standard deviation) relative to the total detected endogenous pool of miRNAs. This represents the background signal for the level of precandidate miRNA relative to the total endogenous pool of miRNAs in this study. While the abundance of mature exogenous miHTTs relative to the total endogenous pool of miRNAs varied greatly across the 16 AAV1-iHtt vectors, spanning a wide, ∼1,100-fold range, 13 of the 16 AAV1-iHtt vectors had a relative abundance of <10%. The mean expression levels of mature exogenous miHTTs from Treatments A and O exceeded the sum of all endogenous miRNA levels in the same striatal tissue sample, which were significantly reduced relative to vehicle, suggesting that these precandidate pri-amiRNAs became dominant in Argonaute loading and overharnessed the endogenous miRNA biogenesis pathway. Although very similar levels of *HTT* mRNA lowering, 29–35% on average, were achieved with Treatments A, J, L, and P and the positive control, the level of mature exogenous miHTT relative to the total endogenous pool of miRNAs varied widely from 0.2% for Treatment P to 143.8% for Treatment A. Treatments J and L and the positive control showed intermediate levels of mature exogenous miHTT relative to the total endogenous pool of miRNAs at 5.7%, 0.8%, and 32.2%, respectively. These results demonstrate the importance of evaluating the extent to which the endogenous miRNA biogenesis pathway is harnessed in selecting a therapeutic pri-amiRNA, in addition to levels of target lowering *in vivo*. The level of mature exogenous miHTT relative to the total endogenous pool of miRNAs at a given level of *HTT* mRNA lowering is a key parameter that provides strong differentiation of different pri-amiRNA candidates *in vivo*, with lower levels of mature exogenous miHTT preferred.

As discussed in detail above, the G/P strand ratio varied substantially across the 16 AAV1-iHtt vectors, spanning a very wide range, from 0.0 to 2384 (Fig. 3C). Twelve of the 16 precandidate miHTTs exhibited G/P strand ratios >10 (Treatments A–C, E, and I–P, as well as the positive control), whereas 4 precandidate miHTTs (Treatments D, F, G, and H) showed G/P strand ratios <3, including 3 (Treatments D, F, and H) with ratios of <0.5, indicating that there were more than twice as many P strands as G strands for these 3 precandidate miHTTs. Thus, the G/P strand ratio is a parameter that provides strong differentiation of different pri-amiRNAs *in vivo*, with higher G/P strand ratios preferred.

When we assessed the precision of 5′ end processing of miHTT guide strands, we observed a batch effect when the two cohorts were compared (Fig. 3D). In the first cohort, the positive control showed 96.8% ± 3.0% (*N* = 3, mean ± standard deviation) of miHTT guides with correct 5′ ends, and six of eight precandidate pri-amiRNAs in this cohort had >90% on average of miHTT guide strands with precise 5′ ends. Only two precandidate pri-amiRNAs (Treatments B and F) exhibited slightly lower percentages of miHTT guide strands with precise 5′ ends (83.2% ± 2.2% and 85.7% ± 21.4%, respectively). In the second cohort, however, none of the precandidate pri-amiRNAs showed mean percentages of more than 90% of miHTT guide strands with precise 5′ ends. Nevertheless, the percentages of correct 5′ end start of the guide strand were in the range from 82.9% ± 0.0% to 89.8% ± 0.3%. These results indicate that the precision of 5′ end processing of miRNA guide strands *in vivo* provides modest differentiation of our precandidate pri-amiRNAs.

### Combinatorial effect of G strand and P strand sequences together with scaffold on pri-amiRNA processing in the YAC128 mouse striatum

As expected, the scaffold was a determinant of pri-amiRNA processing. Different scaffolds with the same G and P strand sequences resulted in different levels of mature exogenous miRNA relative to the total endogenous pool of miRNAs (Fig. 3B) and different G/P strand ratios (Fig. 3C) in the YAC128 mouse striatum. For example, Treatments A and M comprised the same G and P strand sequences inserted into different scaffolds and within the context of each of these scaffolds exhibited highly distinct levels of mature exogenous miRNA relative to the total endogenous pool of miRNAs (143% for Treatment A vs. 1.8% for Treatment M) and different G/P strand ratios (423 for Treatment A vs. 135 for Treatment M).

However, the scaffold was not the sole determinant of pri-amiRNA processing. There was a dramatic interaction of the G and P strand sequences with the scaffold that resulted in a given scaffold producing very different levels of mature miRNA relative to the total endogenous pool of miRNAs and G/P strand ratios, depending on the specific G and P strand sequences within the scaffold. For example, although Treatments A and B comprised the same scaffold but contained different G and P strand sequences inserted into this scaffold, the level of mature exogenous miRNA relative to the total endogenous pool of miRNAs was 143% for Treatment A but only 0.1% for Treatment B. In addition, the G/P strand ratio for Treatment A was 423 but only 24 for Treatment B. As another example, Treatments D and J comprised the same scaffold, which was different from that of Treatments A and B, with different G and P sequences inserted into the scaffold. The level of mature exogenous miRNA relative to the total endogenous pool of miRNAs was substantially lower for Treatment D (0.1%) than for Treatment J (5.7%), and the G/P strand ratio was much higher for Treatment J (629) than for Treatment D (0.3). These results demonstrate the dramatic impact of the G and P strand sequences on pri-amiRNA processing for a given scaffold and support a combinatorial effect of scaffold and G and P strand sequences on pri-amiRNA processing.

### Pri-amiRNA processing efficiency and precision in YAC128 mouse striatum is similar to *in vitro* pri-amiRNA processing

A comparison of pri-amiRNA processing in HeLa cells and neuronally differentiated SH-SY5Y cells *in vitro* versus YAC128 mice *in vivo* shows excellent concordance of the relative level of mature exogenous miHTT relative to the total endogenous pool of miRNAs, relative G/P strand ratios, and precision of 5′ end processing of miHTT guide strands.

The abundance of mature exogenous miHTTs relative to the total endogenous pool of miRNAs spanned a wide range in YAC128 mouse striatum (Fig. 3B), HeLa cells (Fig. 2B), and neuronally differentiated SH-SY5Y cells (Fig. 2B). Treatment K exhibited the lowest relative levels of 0.2% and 0.4% in neuronally differentiated SH-SY5Y and HeLa cells, respectively, and <0.05% *in vivo*, whereas Treatment A exhibited the highest relative levels of 168.2% and 200.8% in neuronally differentiated SH-SY5Y and HeLa cells, respectively, and 143.8% *in vivo*. Treatment O also exhibited very high relative levels, similar to Treatment A both *in vitro* and *in vivo*. In general, the treatments that exhibited low levels of precandidate miHTTs relative to the total endogenous pool of miRNAs *in vitro* also showed relatively low levels in YAC128 mouse striatum. For example, Treatments B, H, K, and P resulted in the lowest relative levels of mature exogenous miHTTs (<1% of the total endogenous miRNA pool) both *in vitro* and *in vivo*. Treatments D and L resulted in the next lowest relative levels of mature exogenous miHTTs both *in vitro* and *in vivo*, with <4% relative levels *in vitro* and <1% relative levels *in vivo*. Treatments C, E, F, G, I, J, M, and N provided intermediate relative levels *in vitro* (5–32%) and in YAC128 mouse striatum (1–11%). Thus, the rank order of precandidate pri-amiRNAs with respect to harnessing the endogenous RNAi pathway in YAC128 mouse striatum was predicted well by their rank order *in vitro*.

For a given precandidate miHTT, the G/P strand ratio differed by 1.5- to 5.1-fold compared across YAC128 mouse striatum (Fig. 3C), HeLa cells (Fig. 2C), and neuronally differentiated SH-SY5Y cells (Fig. 2C). In both YAC128 mice striatum and the two *in vitro* systems, G/P strand ratios were very high (>100) for Treatments A, C, E, I, J, M, and N (except that for Treatment N, the G/P strand ratio was 97.9 in HeLa cells), high (8–100) for Treatments B, K, L, and P, and low (<3) for Treatments D, F, G, and H. For Treatment O, the G/P strand ratio was 163.1 in YAC128 mouse striatum, 74.7 in HeLa cells, and 56.3 in neuronally differentiated SH-SY5Y cells.

The precision of 5′ end processing of the miHTT guide strand spanned a similar range in YAC128 mouse striatum, HeLa cells, and neuronally differentiated SH-SY5Y cells, ranging from 73% to 98% *in vitro* (Fig. 2D) and 83% to 98% *in vivo* (Fig. 3D). Treatments B and F were consistently on the lower end of these ranges in all three test systems. In contrast, other treatments such as Treatment O exhibited 94.5% and 92.1% precision of 5′ end processing of the miHTT guide strand in HeLa and neuronally differentiated SH-SY5Y cells, respectively, but only 82.9% precision of 5′ end processing of the miHTT guide strand in YAC128 mouse striatum.

### Importance of efficient and precise pri-amiRNA processing in addition to target lowering in YAC128 mice and *in vitro* systems for selecting precandidates to evaluate in NHP

The *in vivo* results from YAC128 mice, taken together, led to our ranking of the precandidate pri-amiRNAs and selecting the subset to move forward into NHP. The requirement for statistically significant lowering of human mutant *HTT* mRNA in the striatum by at least 20% resulted in 7 of the 16 precandidate pri-amiRNAs remaining under consideration: Treatments A, B, F, J, L, M, and P. Treatments A, F, and J produced relatively high utilization of the endogenous RNAi pathway (5.7–143.8%) and thus were eliminated from further consideration. In addition, Treatment F exhibited a G/P strand ratio of <0.05, indicating a relatively high proportion of P strands generated relative to G strands, further supporting its removal from further consideration. Given the capacity to evaluate three of these AAV1-iHtt precandidate vectors in an NHP study, and the lower G/P strand ratio and consistently lower precision of 5′ end processing of the miHTT guide strand for Treatment B, Treatments L, M, and P were prioritized for the NHP study to select the lead candidate.

### Precandidate AAV1-iHtt vectors are generally well tolerated in NHP

New batches of AAV1-iHtt vectors (AAV1-iHtt-1 corresponding to Treatment P, AAV1-iHtt-2 corresponding to Treatment M, and AAV1-iHtt-3 corresponding to Treatment L) were produced for the NHP study. Biochemical and biophysical characterization of these batches included titer, relative purity of the capsid proteins, the percentage of full capsids, aggregation and endotoxin levels, as well as genome integrity (Supplementary Fig. S5). Before conducting the study in NHP, the pharmacological activities of these newly produced batches of AAV1-iHtt vectors were confirmed in YAC128 mice (Supplementary Fig. S6). Human *HTT* mRNA reduction was evaluated by RT-qPCR, using mouse XPNPEP1 as the endogenous reference mRNA. Animals were necropsied at 4 weeks following bilateral intrastriatal injection of AAV1-iHtt-1 (corresponding to Treatment P), AAV1-iHtt-2 (corresponding to Treatment M), or AAV1-iHtt-3 (corresponding to Treatment L) at a dose of 3.6 × 10^10^ VG per animal, with seven to eight animals per group, and striatum punches were collected. As expected, significant *HTT* mRNA reduction in the striatum was observed for each of the three AAV1-iHtt vectors relative to the vehicle group (44%, 42%, and 43% lowering for AAV1-iHtt-1, AAV1-iHtt-2, and AAV1-iHtt-3, respectively, compared with 35% *HTT* mRNA lowering for the positive control).

NHPs with no detectable or low levels of anti-AAV1 NAb activity in the serum underwent acute neurosurgical infusion of AAV1-iHtt-1, AAV1-iHtt-2, and AAV1-iHtt-3 into the putamen at a dose of 9.00 × 10^10^ (one putamen in each of six animals) or 2.70 × 10^11^ VG (one putamen in each of six animals) or vehicle (both putamens in each of three animals). All animals were followed for 36 ± 3 days. Assessment of MRI images collected during infusion procedures indicates that all infusions were successfully placed within the left and right putamen.

Postoperative assessments, including daily health observations, body weights, food consumption, and clinical pathology, all indicated that there were no significant alterations in the animals' health. There were no abnormalities observed in any of the major organs during gross necropsy. Importantly, there were no histopathologic findings in the putamen that were considered adverse, based on hematoxylin and eosin staining. In general, observations such as minimal to mild gliosis were noted in all groups, including the vehicle control, and were consistent with injection into the putamen and a needle track. In addition, administration of AAV was associated with minimal to mild mononuclear cell infiltrates. Thus, intraputaminal administration of AAV1-iHtt-1, AAV1-iHtt-2, and AAV1-iHtt-3 was generally well tolerated in NHP over the 5-week in-life period.

### Dose-dependent lowering of *HTT* mRNA in NHP putamen by precandidate AAV1-iHtt vectors

**Figure 4.**
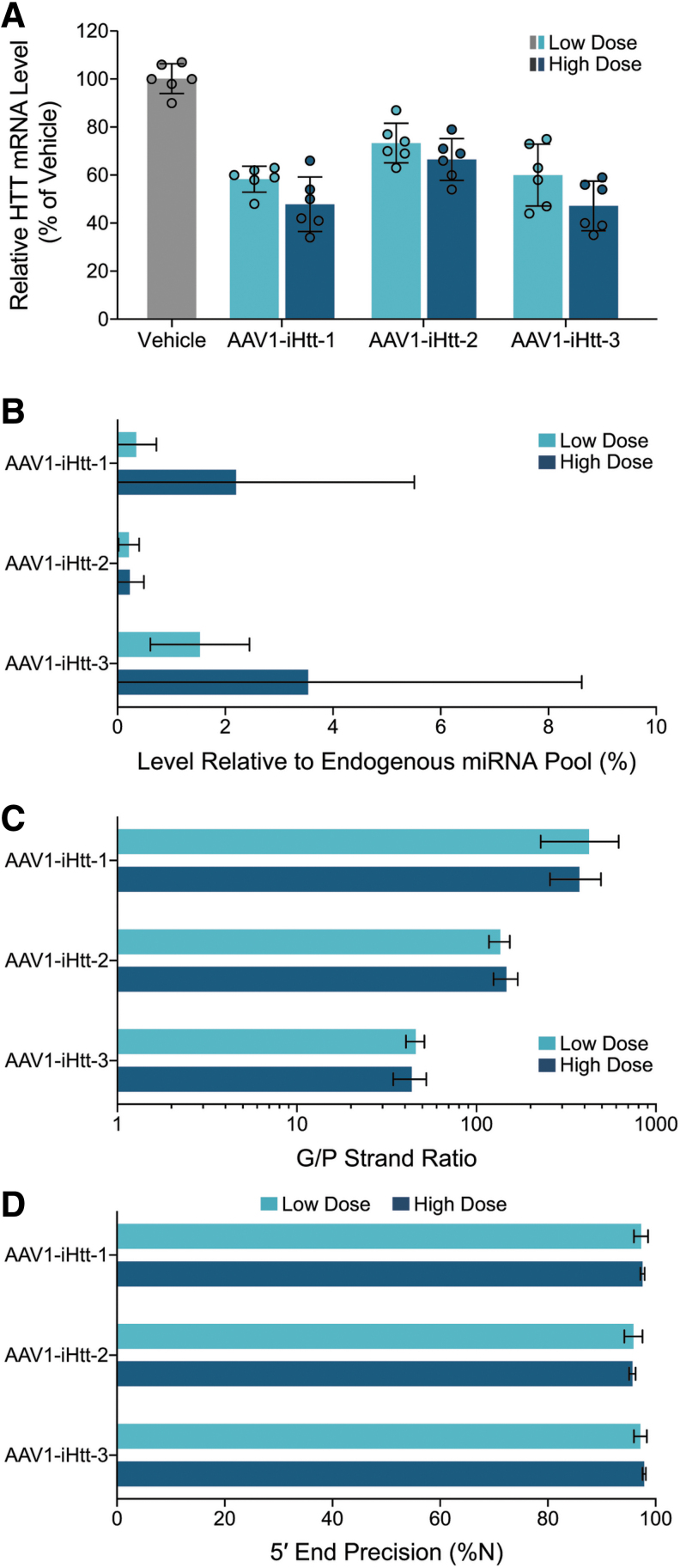
*HTT* mRNA lowering and pri-amiRNA processing in NHP putamen 5 weeks after intraputaminal administration of low (9 × 10^10^ VG per putamen) or high (2.7 × 10^11^ VG per putamen) dose of AAV1-iHtt-1, AAV1-iHtt-2, or AAV1-iHtt-3. **(A)** Relative *HTT* mRNA levels in the NHP putamen. Tissue punches were measured by the bDNA assay for *HTT*, TBP, AARS, and XPNPEP1 mRNA levels. *HTT* mRNA levels were normalized to the geometric mean of the mRNA levels of the three housekeeping genes TBP, AARS, and XPNPEP1 and subsequently compared to the average normalized *HTT* mRNA level of the vehicle group. Each symbol represents the average relative *HTT* mRNA level in the ten punches from one putamen. *N* = 6 per AAV treatment per dose, *N* = 6 for vehicle. **(B–D)** Pri-amiRNA processing in the NHP putamen was evaluated by small RNA sequencing. *N* = 6 per AAV treatment group, one punch per putamen. **(B)** Level of mature exogenous miHTT relative to the total endogenous pool of miRNAs. **(C)** Ratio of guide to passenger strands of the mature exogenous miHTT. **(D)** Percentage of mature exogenous miHTT guide strands with precise 5′ ends. For all *panels*, the group mean ± standard deviation is shown for each treatment. AARS, alanyl-tRNA synthetase; bDNA, branched DNA; TBP, TATA-box binding protein. Color images are available online.

To evaluate *HTT* mRNA reduction in NHP putamen after intraputaminal injection of precandidate AAV1-iHtt vectors, animals were necropsied at 5 weeks following administration, and ten punches were collected from each putamen. *HTT* transcript levels were assessed using a bDNA assay for *HTT* mRNA and the endogenous reference mRNAs TBP, AARS, and XPNPEP1. Each of the three AAV1-iHtt vectors resulted in significant *HTT* mRNA reduction in the putamen relative to the vehicle group that was dose dependent (two-way ANOVA with Tukey's multiple comparisons *post hoc* test, *p* < 0.05; Fig. 4A). At the high dose (2.7 × 10^11^ VG per putamen), there was 52%, 33%, and 53% *HTT* mRNA lowering on average for AAV1-iHtt-1, AAV1-iHtt-2, and AAV1-iHtt-3, respectively, whereas at the low dose (9 × 10^10^ VG per putamen), there was 42%, 27%, and 40% *HTT* mRNA lowering on average, respectively. A two-way ANOVA main effects model with Tukey's multiple comparisons test was used and showed a statistically significant difference both by dose (*F* = 10.68, *p* = 0.0023) and by vector treatment (*F* = 31.89, *p* < 0.0001). There was significantly more reduction of *HTT* mRNA with AAV1-iHtt-1 and AAV1-Htt-3 than with AAV1-iHtt-2 at either the low or high dose (*p* < 0.01). However, there was no significant difference between AAV1-iHtt-1 and AAV1-iHtt-3 in reducing *HTT* mRNA at either dose level. The high dose of AAV1-iHtt-2 achieved the same level of *HTT* mRNA lowering as a threefold lower dose of AAV1-iHtt-1 or AAV1-iHtt-3. These results indicate that AAV1-iHtt-1 and AAV1-iHtt-3 are more potent than AAV1-iHtt-2 in lowering *HTT* mRNA in the NHP putamen.

The importance of sufficient tissue sampling is illustrated in scatter plots of data from all punches (Supplementary Fig. S7). Substantial variation in *HTT* mRNA lowering was observed across the 10 putamen punches from a given animal at a given dose. This variability in *HTT* mRNA lowering across putamen punches is likely due to the variability in vector distribution within the putamen (as reflected in the variation in VG copies/diploid genome across putamen samples, shown in Supplementary Fig. S8B) and the relatively small size of the tissue punches from the putamen which reveal and highlight this heterogeneity. Of a total of 60 putamen punches analyzed per AAV1-iHtt dose group, the percentage of putamen punches with at least 30% *HTT* mRNA reduction at the high dose was 80%, 52%, and 77% for AAV1-iHtt-1, AAV1-iHtt-2, and AAV1-iHtt-3, respectively, whereas the percentage of putamen punches with at least 30% *HTT* mRNA reduction at the low dose was 58%, 45%, and 62%, respectively.

Thus, the pharmacological potency for *HTT* mRNA reduction is comparable for AAV1-iHtt-1 and AAV1-iHtt-3, and greater than that for AAV1-iHtt-2, based on the dose levels evaluated.

### Dose-dependent levels of VGs in NHP putamen after administration of precandidate AAV1-iHtt vectors

To quantify VG copy numbers in NHP putamen after intraputaminal injection of AAV1-iHtt vectors, whole cell DNA was purified from aliquots of lysates from the tissue punches used for mRNA measurements and were subsequently evaluated by ddPCR. Each of the three AAV1-iHtt vectors resulted in substantial numbers of VG copies in the putamen (Supplementary Fig. S8A): 272 and 517 VG copies/diploid genome for AAV1-iHtt-1, 230 and 527 VG copies/diploid genome for AAV1-iHtt-2, and 264 and 554 VG copies/diploid genome for AAV1-iHtt-3, at low and high dose levels, respectively, on average. VG copy number levels were below the lower level of quantification (BLLOQ) for most putamen punches from the vehicle group. When the BLLOQ samples were assigned a value of 0 and then a mean for each animal determined, the averages for the vehicle groups corresponding to the low and high dose ddPCR runs were 0 and 0.1 VG copies/diploid genome, respectively.

For all three AAV1-iHtt vectors tested, VG copy numbers were dose dependent but less than dose proportional. There was no significant difference in VG copy numbers between AAV1-iHtt-1, AAV1-iHtt-2, and AAV1-iHtt-3 at the low dose, nor at the high dose (one-way ANOVA with Tukey's multiple comparisons test).

To evaluate the relationship between VG level and *HTT* mRNA lowering in the putamen, the relative levels of remaining *HTT* mRNA were plotted as a function of VG levels (Supplementary Fig. S8B). The number of VG copies detected for each vector correlated with *HTT* mRNA lowering in the putamen, with more VG copies per diploid genome resulting in more *HTT* mRNA lowering. Since the data appeared sigmoidal, a nonlinear regression model comprising a four-parameter curve fit was used, with the maximum and minimum asymptotes constrained at 100% and 20%, respectively. The effective concentration for producing 50% of the maximal response relative to the minimum level (EC_50_)'s for *HTT* mRNA lowering calculated from these curve fits represent relative *HTT* mRNA levels of 60%, midway between the two asymptotes. These EC_50_'s suggest that AAV1-iHtt-1 and AAV1-iHtt-3 are more potent than AAV1-iHtt-2, in that lower VG levels are required for *HTT* mRNA lowering for AAV1-iHtt-1 and AAV1-iHtt-3 than for AAV1-iHtt-2. Specifically, ∼40% *HTT* mRNA suppression was achieved with 40 VG copies/diploid genome after treatment with AAV1-iHtt-1, 273 VG copies/diploid genome after treatment with AAV1-iHtt-2, and 68 VG copies/diploid genome after treatment with AAV1-iHtt-3. Spearman correlation coefficients were −0.8968, −0.9087, and −0.8783 for the AAV1-iHtt-1, AAV1-iHtt-2, and AAV1-iHtt-3 curves, respectively, indicating strong negative correlations between relative *HTT* mRNA levels and VG levels for all three AAV1-iHtt vectors.

### Pri-amiRNA processing in NHP putamen after administration of precandidate AAV1-iHtt vectors is precise and efficient

Putamen samples from the same NHPs used to evaluate *HTT* mRNA lowering and VG levels were collected and submitted for small RNA deep sequencing. The 18 samples from the high dose groups had 23.1–39.6 million raw reads with the correct adapter sequence per sample, whereas the 18 samples from the low dose groups had 14.8–37.8 million raw reads with the correct adapter sequence per sample. In the vehicle group, there were 15.9–41.8 million raw reads with the correct adapter sequence per sample. These data indicate that all samples showed sufficient sequencing depth for evaluation of pri-amiRNA processing.

To evaluate endogenous miRNA profiles in putamen samples following AAV1-iHtt administration, the percentage of reads aligned to annotated rhesus miRNA hairpin sequences was assessed. In the vehicle group, an average of 54.1% of reads (range 47.7–61.1%) was aligned to annotated rhesus miRNA hairpin sequences (data not shown) and, thus, represented endogenous miRNAs; this served as the baseline endogenous miRNA profile in this study. In animals treated with AAV1-iHtt-1, AAV1-iHtt-2, or AAV1-iHtt-3 at either high or low dose, 53.4–55.8% of reads were aligned to endogenous rhesus miRNA hairpin sequences (data not shown), similar to the baseline of 54.1% from the vehicle group. These results suggest that the endogenous miRNA profiles for all putamen samples following AAV1-iHtt-1, AAV1-iHtt-2, or AAV1-iHtt-3 administration were not affected by treatment.

The level of mature exogenous miHTT relative to the total endogenous pool of miRNAs was evaluated (Fig. 4B). For the three animals (two putamen punches per animal) in the vehicle group, the detectable miHTT read number (with all three precandidate miRNAs used as reference) was <10 rpm, resulting in expression levels of 0% ± 0.00% relative to the total detected endogenous pool of miRNAs. As with the small RNA sequencing conducted on YAC128 mouse striatal samples, this represents the background signal for the level of precandidate miHTT relative to the total endogenous pool of miRNAs in this study. In NHPs treated with AAV1-iHtt-1 or AAV1-iHtt-3, but not AAV1-iHtt-2, the relative mean putaminal expression level of mature exogenous miHTT was dose related. At the low dose, the level of miHTT relative to the total endogenous pool of miRNAs was approximately fivefold lower for AAV1-iHtt-1 (0.35% on average) than AAV1-iHtt-3 (1.53% on average), indicating that AAV1-iHtt-1 treatment achieved a similar level of *HTT* mRNA lowering as AAV1-iHtt-3 at this dose, but with substantially less harnessing of the endogenous miRNA biogenesis pathway. The high dose of AAV1-iHtt-2 achieved a similar level of *HTT* mRNA lowering as the low dose of AAV1-iHtt-1 (Fig. 4A) with a comparable level of precandidate miHTT relative to the total endogenous pool of miRNAs. Thus, the level of mature exogenous miHTT relative to the total endogenous pool of miRNAs at a given dose and at a given level of *HTT* mRNA lowering is a parameter that differentiates these AAV1-iHtt vectors.

For each AAV1-iHtt vector, the G/P strand ratio was similar across the two dose levels administered (Fig. 4C). However, the G/P strand ratio varied substantially across AAV1-iHtt vectors. AAV1-iHtt-1 showed the highest G/P ratios (423.8 and 375.5 on average at the low and high doses, respectively), which were ∼10-fold higher than the G/P strand ratios for AAV1-iHtt-3 (46.0 and 43.6 on average at the low and high doses, respectively). The G/P strand ratios for AAV1-iHtt-2 were intermediate between those for AAV1-iHtt-1 and AAV1-iHtt-3. Thus, the G/P strand ratio is a parameter that differentiates these AAV1-iHtt vectors in NHP putamen, with AAV1-iHtt-1 exhibiting the most favorable G/P strand ratio.

The precision of 5′ end processing of miHTT guide strands was assessed. All three AAV1-iHtt vectors at the two dose levels administered showed high mean percentages (95–98%) of miHTT guide strands with precise 5′ ends (Fig. 4D), indicating that the 5′ end of the miHTT guide strands is primarily processed correctly. In addition, these results indicate that the precision of 5′ end processing of miHTT guide strands was very similar across the three AAV1-iHtt vectors, as well as across the two dose levels evaluated. Thus, the precision of 5′ end processing of miHTT guide strands does not differentiate these AAV1-iHtt vectors in NHP putamen.

### Pri-amiRNA processing of three top precandidates in NHP putamen differs from *in vitro* cells and YAC128 mouse striatum

A comparison of pri-amiRNA processing of precandidates L (AAV1-iHtt-3), M (AAV1-iHtt-2), and P (AAV1-iHtt-1) in NHP putamen, YAC128 mouse striatum, HeLa cells, and neuronally differentiated SH-SY5Y cells shows excellent concordance of relative precision of 5′ end processing of miHTT guide strands (Table 1), given the RNA extraction and small RNA library preparation in different laboratories with different methods. Levels of mature exogenous miHTT relative to the total endogenous pool of miRNA were fairly similar in NHP putamen versus YAC128 striatum across the three candidates evaluated in NHP (Table 1). However, *in vitro* (Table 1), one of the three precandidates (Treatment M, corresponding to AAV1-iHtt-2) showed substantially higher levels of mature exogenous miHTT relative to the total endogenous miRNA pool, indicating that *in vitro* systems do not always predict this parameter well in YAC128 mice or in NHP. In addition, G/P strand ratios differed substantially in NHP putamen versus YAC128 mouse striatum and neuronally differentiated SH-SY5Y cells (Table 1) for one of the three precandidates (Treatment P, corresponding to AAV1-iHtt-1), indicating that neither *in vitro* systems nor YAC128 mice reliably predict this parameter well in NHP.

**Table 1.** Comparison of primary artificial microRNA processing across cells in vitro, YAC128 mouse striatum, and nonhuman primate putamen

	P (AAV1-iHtt-1)			M (AAV1-iHtt-2)			L (AAV1-iHtt-3)		
	Level Relative to Endogenous miRNA Pool (%)	G/P Strand Ratio	5′ End Precision (%* N*)	Level Relative to Endogenous miRNA Pool (%)	G/P Strand Ratio	5′ End Precision (%* N*)	Level Relative to Endogenous miRNA Pool (%)	G/P Strand Ratio	5′ End Precision (%* N*)
HeLa^a^	0.7^b^	8.8^b^	96.4^b^	27.9	130.6	96.3	1.4^b^	23^b^	98^b^
SH-SY5Y^a^	0.6	70.7	94.6	9.2	126.3	93.6	3.6	38.3	95.3
YAC128 striatum^c^	0.2 ± 0.0	39.9 ± 3.8	86.8 ± 0.4	1.8 ± 2.2	135.3 ± 18.8	87.1 ± 1.1	0.8 ± 0.6	82.9 ± 10.6	89.8 ± 0.3
NHP putamen (low)^d^	0.35 ± 0.37	423.8 ± 195.9	97.3 ± 1.3	0.21 ± 0.19	135.7 ± 18.1	95.9 ± 1.7	1.53 ± 0.92	46.0 ± 5.4	97.2 ± 1.2
NHP putamen (high)^d^	2.20 ± 3.31	375.5 ± 118.4	97.6 ± 0.4	0.23 ± 0.26	147.3 ± 22.7	95.7 ± 0.6	3.54 ± 5.08	43.6 ± 9.1	97.9 ± 0.3

Pri-amiRNA processing was evaluated after transduction with treatment P, M, or L, or correspondingly AAV1-iHtt-1, AAV1-iHtt-2, or AAV1-iHtt-3, respectively. Small RNA sequencing was carried out to assess level of mature exogenous miHTT relative to the total endogenous pool of miRNAs, ratio of guide to passenger strands of the mature exogenous miHTT, and percentage of mature exogenous miHTT guide strands with precise 5′ ends.

^a^ HeLa and neuronally differentiated SH-SY5Y cells were transduced with AAV2-packaged pri-amiRNAs targeting *HTT* mRNA at a MOI of 1.0 × 10^5^ VG per cell and harvested approximately 24–48 h later. *N* = 1 per treatment.

^b^ Pri-amiRNA processing results obtained after plasmid transfection.

^c^ Pri-amiRNA processing in YAC128 mouse striatum 4 weeks after bilateral intrastriatal injection of AAV1-packaged pri-amiRNAs targeting HTT at a dose of 2.6 × 10^10^ to 6.0 × 10^10^ VG per animal. *N* = 3 per AAV treatment group. The group mean ± standard deviation is shown for each treatment.

^d^ Pri-amiRNA processing in NHP putamen 5 weeks after intraputaminal administration of low (9 × 10^10^ VG per putamen) or high (2.7 × 10^11^ VG per putamen) dose of AAV1-iHtt-1, AAV1-iHtt-2, or AAV1-iHtt-3. *N* = 6 per AAV treatment group, one punch per putamen. The group mean ± standard deviation is shown for each treatment.

AAV, adeno-associated viral; AAV1, AAV serotype 1; AAV2, AAV serotype 2; G, guide; G/P, guide-to-passenger; *HTT*, huntingtin; miRNA, microRNA; MOI, multiplicity of infection; NHP, nonhuman primate; P, passenger; pri-amiRNA, primary artificial microRNA; VG, vector genome.

The precision of 5′ end processing of miHTT guide strands was high (>87%) and indistinguishable across the vectors AAV1-iHtt-1, AAV1-iHtt-2, and AAV1-iHtt-3 in NHP putamen at the low and high doses, in YAC128 mouse striatum, and in HeLa and neuronally differentiated SH-SY5Y cells. In addition, the absolute precision of 5′ end processing of miHTT guide strands was consistent across NHP putamen (95–98%), YAC128 mouse striatum (87–90%), and *in vitro* systems (93–97%), given the relatively lower 5′ end precision of miHTT guide strands measured in the corresponding entire cohort of YAC128 mouse striatum samples. Thus, in all systems evaluated—NHP putamen, YAC128 mouse striatum, HeLa cells, and neuronally differentiated SH-SY5Y cells—the 3 AAV vectors were indistinguishable with respect to the precision of 5′ end processing of miHTT guide strands.

The level of mature miHTT relative to the total endogenous pool of miRNAs was <4% in NHP putamen at both dose levels and in YAC128 striatum. At doses that provided the same degree of *HTT* mRNA lowering in NHP putamen (low dose of AAV1-iHtt-1, high dose of AAV1-iHtt-2, and low dose of AAV1-iHtt-3), AAV1-iHtt-1 and AAV1-iHtt-2 harnessed the endogenous RNAi pathway to a lower extent on average than AAV1-iHtt-3. In YAC128 mouse striatum, AAV1-iHtt-1 harnessed the endogenous pathway to the lowest extent on average followed by AAV1-iHtt-3 and then AAV1-iHtt-2. Taken together, these *in vivo* data from NHP putamen and YAC128 striatum suggest that AAV1-iHtt-1 harnesses the endogenous pathway to the lowest extent among the 3 AAV1-iHtt vectors. Similarly, in HeLa and neuronally differentiated SH-SY5Y cells, Treatments P and L (which correspond to AAV1-iHtt-1 and AAV1-iHtt-3, respectively) exhibited low levels of mature exogenous miHTT relative to the total endogenous pool of miRNA. However, in contrast, Treatment M (which corresponds to AAV1-iHtt-2) exhibited noticeably higher relative levels of mature exogenous miHTT *in vitro* (27.9% and 9.2% in HeLa and neuronally differentiated SH-SY5Y cells, respectively) than *in vivo* (1.8%).

The relative and absolute G/P strand ratios for the precandidate pri-amiRNAs in NHP putamen contrasted substantially with these ratios in YAC128 mouse striatum and in neuronally differentiated SH-SY5Y cells, especially for one of the precandidate pri-amiRNAs. For AAV1-iHtt-2, the average G/P strand ratios were indistinguishable, comprising 135.7, 147.3, 135.3, and 126.3 for NHP putamen at low dose, NHP putamen at high dose, YAC128 striatum, and neuronally differentiated SH-SY5Y cells, respectively. However, for AAV1-iHtt-1, the average G/P strand ratios were ∼6- to 10-fold higher in NHP putamen (423.8 and 375.5 at low and high doses, respectively) than in YAC128 striatum (39.9) and neuronally differentiated SH-SY5Y cells (70.7). In contrast, for AAV1-iHtt-3, the average G/P strand ratios were approximately twofold lower in NHP putamen (46.0 and 43.6 at low and high doses, respectively) and neuronally differentiated SH-SY5Y cells (38.3) than in YAC128 striatum (82.9). Thus, for AAV1-iHtt-1, the G/P strand ratio in NHP putamen was substantially superior to either neuronally differentiated SH-SY5Y cells or YAC128 striatum, whereas for AAV1-iHtt-2 and AAV1-iHtt-3, the G/P strand ratios in NHP putamen, neuronally differentiated SH-SY5Y cells, and YAC128 striatum were more similar.

### Selection of optimized pri-amiRNA based on efficient and precise pri-amiRNA processing, in addition to *HTT* lowering and general tolerability

Taken together, the *in vivo* results in NHP putamen that comprised *HTT* mRNA lowering and pri-amiRNA processing were the primary basis of selecting the pri-amiRNA sequence in AAV1-iHtt-1 as the optimized pri-amiRNA for the treatment of HD. At the doses used, AAV1-iHtt-1 showed target levels of *HTT* mRNA lowering in the NHP putamen of 40% and 53% at the low and high doses, respectively, concomitant with: (A) minimal harnessing of the endogenous miRNA biogenesis pathway (0.35% and 2.20% at the low and high doses, respectively), (B) a high G/P strand ratio (423.8 ± 195.9 and 375.5 ± 118.4 at the low and high doses, respectively), and (C) precise 5′ end processing of the miHTT G strand (97.3% and 97.6% at the low and high doses, respectively). Furthermore, the dose levels used for the 5-week NHP study were well tolerated based on daily health observations, body weights, food consumption, clinical pathology, and lack of abnormalities in any of the major organs during gross necropsy. In addition, the NHP results were supported by YAC128 mouse and *in vitro* studies that demonstrated similar robust *HTT* mRNA lowering accompanied by efficient and precise processing of the pri-amiRNA to the miHTT G strand without perturbing the endogenous miRNA pool.

## Discussion

For AAV-mediated RNAi gene therapy, efficient and precise processing of the expressed miRNA precursor to the mature therapeutic guide strand is a critical determinant of potency and safety.^13,14,54^ In this study, we conducted a systematic comparison of pri-amiRNA processing of a number of AAV-pri-amiRNAs across multiple *in vitro* and *in vivo* systems, including mice and NHPs. Our results inform the similarities and differences between *in vitro* and *in vivo* systems and, in particular, the predictivity of *in vitro* for *in vivo* results and of mouse for NHP results on key attributes. These attributes include level of mature exogenous miRNA relative to the total endogenous pool of miRNAs, G/P strand ratio, and accuracy of 5′ end guide strand processing. Our findings impact the future selection and use of these systems for evaluating different pri-amiRNAs. Moreover, our results highlight the combinatorial impact of scaffold, guide, and passenger strand sequences on pri-amiRNA processing, with implications for both design and testing of pri-amiRNAs *in vitro* and *in vivo*.

Our data demonstrate that: (1) target lowering across multiple cell lines, rather than a single cell line, is preferable for comparing different pri-amiRNAs; (2) among pri-amiRNAs that provide sufficient target lowering *in vitro*, the key pri-amiRNA processing parameters for differentiation *in vitro* are: abundance of mature exogenous miRNA relative to the total endogenous pool of miRNAs, G/P strand ratio, and accuracy of 5′ end guide strand processing; (3) *in vivo* target lowering in the mouse is a more sensitive differentiator of pri-amiRNAs than target lowering *in vitro*; (4) among pri-amiRNAs that provide sufficient target lowering in the mouse, important pri-amiRNA processing parameters for differentiation are the same as those *in vitro* (abundance of mature exogenous miRNA relative to the total endogenous pool of miRNAs, G/P strand ratio, and accuracy of 5′ end guide strand processing) with the rank order of precandidate pri-amiRNAs predicted well by *in vitro* results; and (5) in the NHP, important parameters for differentiation are the same as those *in vitro* and in the mouse—target lowering and pri-amiRNA processing (abundance of mature exogenous miRNA relative to the total endogenous pool of miRNAs, G/P strand ratio, and accuracy of 5′ end guide strand processing)—with abundance of mature exogenous miRNA relative to the total endogenous pool of miRNAs, but not G/P strand ratio, predicted well by mouse or *in vitro* results.

To identify an effective pri-amiRNA for target lowering, our results highlight the importance of conducting an *in vivo* screen. Although our evaluation of 16 precandidate pri-amiRNAs in 3 human cell lines (HeLa, U251MG, and neuronally differentiated SH-SY5Y cells) for lowering of endogenous *HTT* mRNA revealed substantial differences between the pri-amiRNAs, the rank order *in vitro* with respect to target lowering did not completely predict the rank order for target lowering *in vivo* in the striatum, an HD-relevant brain region. In addition, the rank order of pri-amiRNAs in lowering endogenous target mRNA varied substantially across different cell lines *in vitro*, underscoring the importance of using multiple cell types *in vitro* when comparing different pri-amiRNAs for target lowering and moving forward with the pri-amiRNAs that perform best across all *in vitro* systems. In NHP, dose-dependent *HTT* mRNA reductions (and VG levels) in the putamen were observed following intraputaminal administration of three precandidate pri-amiRNAs that had exhibited favorable target lowering in YAC128 mouse striatum and *in vitro*. Thus, with intraparenchymal routes of administration and based on tissues evaluated from the sites of administration, target lowering in NHP mediated by pri-amiRNAs packaged in AAV appears to be predicted well by the mouse.

A high G/P strand ratio is achieved by maximizing production of the mature G strand with minimal levels of the P strand, through optimization of the pri-amiRNA scaffold and miRNA in tandem. High G/P strand ratios result in negligible quantities of the P strand at doses that provide pharmacologically relevant levels of the G strand, thus minimizing any potential off-target pharmacological activity associated with the P strand at therapeutic dose levels. Moreover, a well-designed P strand that does not have complete or nearly complete matches with potential off-targets, together with a high G/P strand ratio, essentially eliminates the off-target pharmacological liability of the P strand.

Our comparison of 16 precandidate pri-amiRNAs showed that the G/P strand ratio differs greatly across these pri-amiRNAs, spanning ranges of ∼3000-fold in 2 cell lines and 2400-fold in YAC128 mouse striatum, with similar rank orders *in vitro* and *in vivo*. For each of our three AAV1-iHtt vectors tested in NHP, the G/P strand ratio in the putamen was similar across the two dose levels administered, as expected. However, G/P strand ratios differed substantially between NHP, the YAC128 mouse, and *in vitro* systems used, indicating that neither these *in vitro* systems nor YAC128 mice reliably predict this parameter in NHP. For AAV1-iHtt-1, the G/P strand ratio was >375 in NHP putamen, illustrating the ability to achieve very high G/P strand ratios with the appropriate pri-amiRNA scaffold and miRNA design. Several naturally occurring scaffolds that utilize the canonical RNAi pathway have been reported to provide effective and safe gene silencing,^16,33,34,44,55^ with miR-33 resulting in the highest G/P strand ratio (1878) for the G and P strands evaluated.^33^ However, based on our findings, different G and P strand sequences inserted into a particular scaffold may result in dramatically altered and possibly suboptimal G/P strand ratios. It remains to be determined whether the miR-33 scaffold provides sufficiently high G/P strand ratios for G and P strands other than those originally evaluated to minimize significant pharmacological contribution of the P strand at therapeutic dose levels.

Since limiting the use of the endogenous miRNA biogenesis pathway is less likely to perturb the endogenous pool of miRNAs and more likely to leave their associated cellular functions intact,^13,14,25^ pri-amiRNAs that produce potent target lowering achieved with lower use of the RNAi pathway are likely to be safer and should be prioritized. Our comparison of 16 precandidate pri-amiRNAs showed that the level of mature exogenous miHTTs relative to the total endogenous pool of miRNAs differs greatly across these pri-amiRNAs, spanning ranges of ∼1000-fold in 2 cell lines and 1,100-fold (from <0.05% to 143.8%) in YAC128 mouse striatum. Although the rank orders were similar *in vitro* and *in vivo*, absolute values observed *in vitro* did not necessarily reflect those in YAC128 mice. Total endogenous miRNA levels in the YAC128 mouse striatum were significantly lower than in the corresponding vehicle group after treatment with the pri-amiRNAs packaged in AAV that resulted in the highest relative levels of mature exogenous miRNA (*e.g*., 143.8% and 119.1% for Treatments A and O, respectively). These results demonstrate that the endogenous miRNA biogenesis pathway can be significantly affected not only by AAV vectors expressing shRNAs^13,14,25,33^ but also by AAV vectors expressing pri-amiRNAs when there is a high level of the mature exogenous miRNA relative to the total endogenous miRNA pool.

Despite the very different levels of the mature exogenous miHTT relative to the total endogenous pool of miRNAs (*e.g*., 143.8% and 0.2% for Treatments A and P, respectively), similar levels of ∼40% *HTT* mRNA lowering were achieved in YAC128 mouse striatum. Thus, the level of mature exogenous miRNA relative to the total endogenous miRNA pool is a key parameter that provides strong differentiation *in vivo* of different pri-amiRNAs packaged in AAV. In selecting a therapeutic pri-amiRNA, it is critical to evaluate both the extent to which the endogenous miRNA biogenesis pathway is harnessed and levels of target lowering *in vivo*. In NHP putamen, endogenous miRNA profiles were not affected by any of our three AAV1-iHtt vectors at either dose level tested, consistent with the relatively low level of mature exogenous miHTTs relative to the total endogenous pool (<4%). In addition, levels of mature exogenous miHTT relative to the total endogenous pool of miRNAs in NHP putamen were similar to those in YAC128 striatum for all three AAV1-iHtt vectors.

To achieve the intended target selectivity of the guide strand, accuracy of 5′-end processing is essential to maintain fidelity of the seed sequence, which is necessary for target mRNA recognition.^56^ Heterogeneity at the 5′ end should be minimized to avoid shifts in the seed sequence and resultant off-target activity. Our comparison of 16 precandidate pri-amiRNAs *in vitro* versus YAC128 mouse striatum shows good precision of 5′ end processing of the guide strand for all pri-amiRNAs, but with some outperforming others. The precision of 5′ end processing of these pri-amiRNAs is similar to that of several naturally occurring scaffolds.^16,33,44,55,57^ In NHP, the precision of 5′ end processing of exogenous miHTT guide strands was high for all three AAV1-iHtt vectors that we tested and, as expected, at both dose levels evaluated.

Differences between *in vitro*, mouse, and NHP test systems with respect to target lowering and G/P strand ratio may be due to variation between species or cell type. The *in vitro* systems we used comprised human cells, whereas our *in vivo* systems comprised mouse and NHP. Cell types *in vitro* included non-neuronal cells (*e.g*., HeLa cells), neuronal cells (neuronally differentiated SH-SY5Y cells), or glial cells (U251MG). The abundance of mature exogenous miHTT relative to the total endogenous pool of miRNAs and G/P strand ratios spanned a very wide range (0.2–200.8 and 0.0–834.4, respectively) but was remarkably consistent across the 16 precandidate pri-amiRNAs in HeLa cells versus neuronally differentiated SH-SY5Y cells. These results suggest that different cell types may similarly process pri-amiRNA candidates driven by a ubiquitous promoter such as CBA and packaged in AAV, with respect to these parameters. *In vivo* samples comprised striatal tissue primarily composed of medium spiny neurons, in contrast to neuronally differentiated SH-SY5Y cells. Nonetheless, levels of mature exogenous miHTT relative to the total miRNA pool were proportionally very similar, and G/P strand ratios were within ∼2-fold of each other in neuronally differentiated SH-SY5Y cells versus YAC128 striatal samples. In contrast, in NHP putamen, G/P strand ratios exhibited different rank orders across the three AAV1-iHtt vectors compared with YAC128 striatum and neuronally differentiated SH-SY5Y cells. Given the importance of tissue and cell type (striatal neurons) and species (NHP), the values for these parameters in NHP putamen were considered to be most relevant for clinical translation. The basis of the observed differences in processing of pri-amiRNA packaged in AAV in NHP putamen versus YAC128 striatum and neuronally differentiated SH-SY5Y cells remains to be determined.

Our results highlight the combinatorial impact of scaffold, G, and P strand sequences on pri-amiRNA processing, with implications for both design and testing of pri-amiRNAs. Strategies focused on scaffold and P strand selection and engineering have been used to optimize pri-amiRNA processing for a given G strand.^14–16,33,44,55,58,59^ Once a G strand sequence is selected, the P strand sequence can be engineered to optimize pri-amiRNA processing. However, the interaction of the G and P strand sequences with the scaffold sequence introduces a significant caveat to extending the findings observed with a given guide strand. Our results show that key processing parameters can be completely altered depending on the particular G and P strand sequences within a given scaffold. Scaffold sequences that are optimal with one pair of G and P strand sequences may not be optimal with a different pair of G and P strand sequences. Thus, a combinatorial approach to scaffold and G and P sequence selection should be used to identify the optimal pri-amiRNA for a target of interest.

From a practical perspective, our results suggest the following approach to optimization and evaluation of pri-amiRNAs for AAV gene therapy. To create libraries of pri-amiRNAs for screening, the scaffold, as well as G and P strand sequences, should be varied in tandem. Testing large numbers of different pri-amiRNAs should be conducted in multiple *in vitro* systems with comparative end points of target lowering and pri-amiRNA processing. The subset of pri-amiRNAs that exhibit sufficient *in vitro* target lowering with the lowest levels of mature exogenous miRNA relative to the total endogenous pool of miRNAs, highest G/P strand ratios, and greatest accuracy of 5′ end G processing should be prioritized to move forward into mouse studies to further narrow the number of precandidate pri-amiRNAs. Based on target lowering and the same pri-amiRNA processing parameters in the mouse, several of these precandidates may then be chosen for final candidate selection in the NHP. In the NHP, these pri-amiRNAs should be assessed for target lowering and the same pri-amiRNA processing parameters to nominate candidate pri-amiRNAs. These candidate pri-amiRNAs should be evaluated for potential clinical benefit with efficacy studies in animal models of disease. Our optimized pri-amiRNAs targeting *HTT* mRNA are undergoing an extensive evaluation of efficacy in the YAC128 mouse model of HD to demonstrate the therapeutic potential of an AAV gene therapy that exhibits efficient and precise pri-amiRNA processing along with potent pharmacological activity for *HTT* mRNA lowering. These efficacy results will be reported elsewhere.

In summary, our results demonstrate a combinatorial effect of G strand, P strand, and scaffold sequences on pri-amiRNA processing, underscoring the critical importance of varying all of these sequences in tandem to optimize pri-amiRNA processing. We provide the first comprehensive comparison of target lowering and pri-amiRNA processing across *in vitro*, rodent, and NHP model systems, using multiple pri-amiRNA candidates that span a wide range of profiles. This comparison highlights the similarities and differences between these model systems and the importance of evaluating not only target lowering but also pri-amiRNA processing *in vitro* and in rodents and large mammals for selecting potent, selective, and safe AAV gene therapies. In particular, key components of such an AAV gene therapy include limiting the use of the endogenous miRNA biogenesis pathway, maximizing the G/P strand ratio with minimal or no detectable P strand activity, and maximizing the precision of 5′ end processing of the G strand, while achieving the target lowering needed for potential therapeutic benefit. Our optimized pri-amiRNA was selected with these considerations, for an AAV gene therapy that exhibits efficient and precise pri-amiRNA processing, along with potent pharmacological activity for *HTT* mRNA lowering and general tolerability *in vivo* for the treatment of HD.

## Supplementary Material

Supplemental data

Supplemental data

Supplemental data

Supplemental data

Supplemental data

Supplemental data

Supplemental data

Supplemental data
