# Supplementary material for: Efficient and Precise Processing of the Optimized Primary Artificial MicroRNA in a Huntingtin-Lowering Adeno-Associated Viral Gene Therapy In Vitro and in Mice and Nonhuman Primates
Source: Hum Gene Ther. 2022 Jan 17;33(1-2):37–60. doi: 10.1089/hum.2021.221 (PMC10112875; doi:10.1089/hum.2021.221)
Supplement: Supplemental data [file Suppl_FigureS1.docx]

**Supplemental Figure S1.** Diagram of recombinant AAV vector which expresses a pri-amiRNA scaffold containing a miRNA targeting human HTT mRNA. ITR, inverted terminal repeat. CBA, chicken β-actin promoter. pA, rabbit beta-globin polyadenylation signal. The miR base-predicted simple secondary structures of the 16 pre-candidate pri-amiRNAs A-P and positive control are shown. Flanking and stem sequences are in black. Guide strand sequences are in pink and red (seed portion). Passenger strand sequences are in blue. Loop sequences are in green. These secondary structures may be more complex than shown (e.g., based on RNA fold).
