# Supplementary material for: Efficient and Precise Processing of the Optimized Primary Artificial MicroRNA in a Huntingtin-Lowering Adeno-Associated Viral Gene Therapy In Vitro and in Mice and Nonhuman Primates
Source: Hum Gene Ther. 2022 Jan 17;33(1-2):37–60. doi: 10.1089/hum.2021.221 (PMC10112875; doi:10.1089/hum.2021.221)
Supplement: Supplemental data [file Suppl_FigureS2.docx]

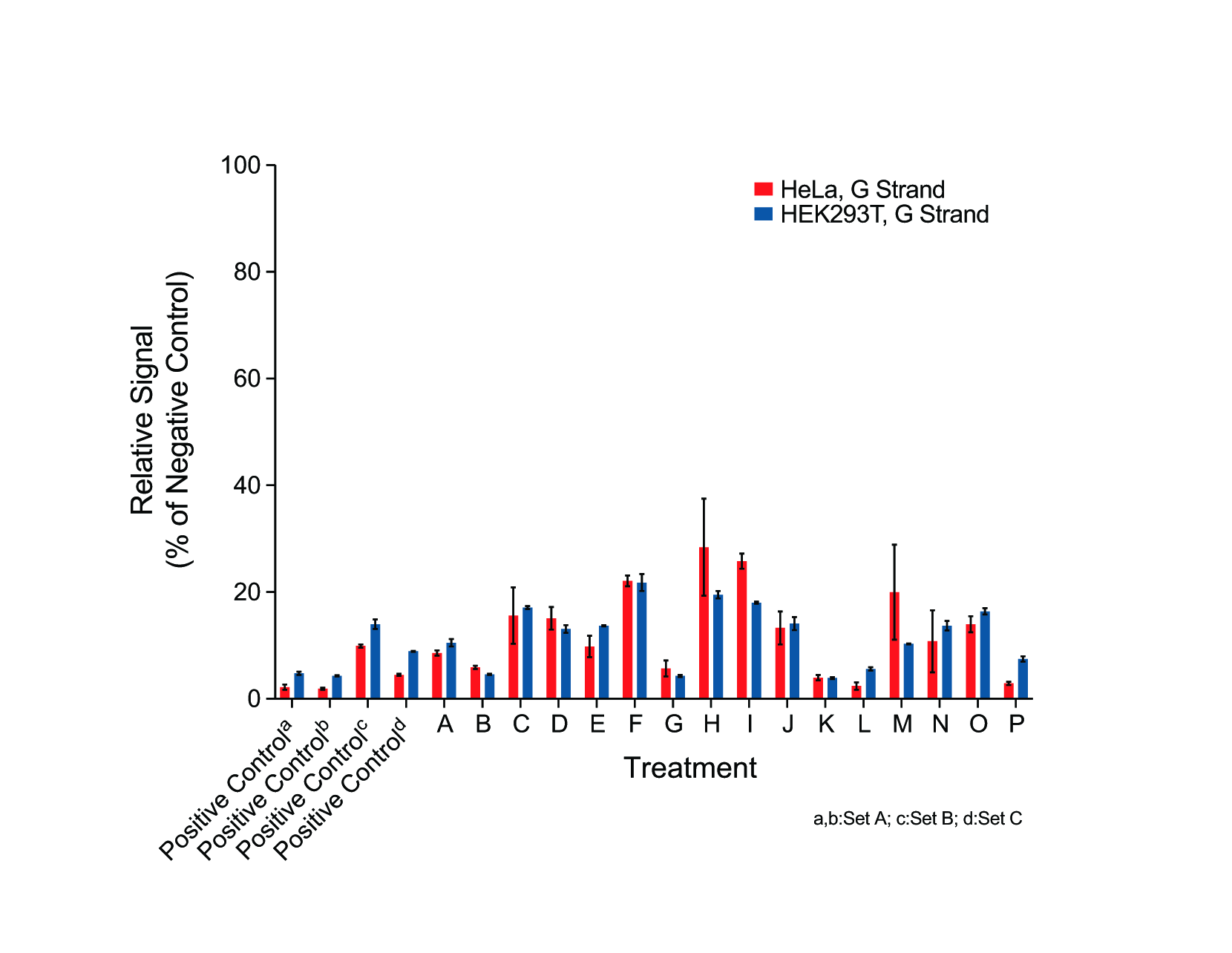


**Supplemental Figure S2.** Reduction of dual-Luc reporter signal in HeLa and HEK293T cells after transfection with treatments A-P. HeLa and HEK293T cells were transfected with HTT pri-amiRNA and guide strand reporter plasmids and lysed for luciferase activity measurements approximately 48 hours later. Sixteen pre-candidate pri-amiRNAs (A-P) were evaluated in 3 different sets, each with positive and negative controls. Set A comprised treatments B and K. Set B comprised treatments A, C-J, and M-O. Set C comprised treatments L and P. Relative reporter signals are expressed as percentage of the negative control signal in that set. The group mean ± standard deviation (*N*=3) is shown for each treatment.
