# Supplementary material for: Efficient and Precise Processing of the Optimized Primary Artificial MicroRNA in a Huntingtin-Lowering Adeno-Associated Viral Gene Therapy In Vitro and in Mice and Nonhuman Primates
Source: Hum Gene Ther. 2022 Jan 17;33(1-2):37–60. doi: 10.1089/hum.2021.221 (PMC10112875; doi:10.1089/hum.2021.221)
Supplement: Supplemental data [file Suppl_FigureS3.docx]

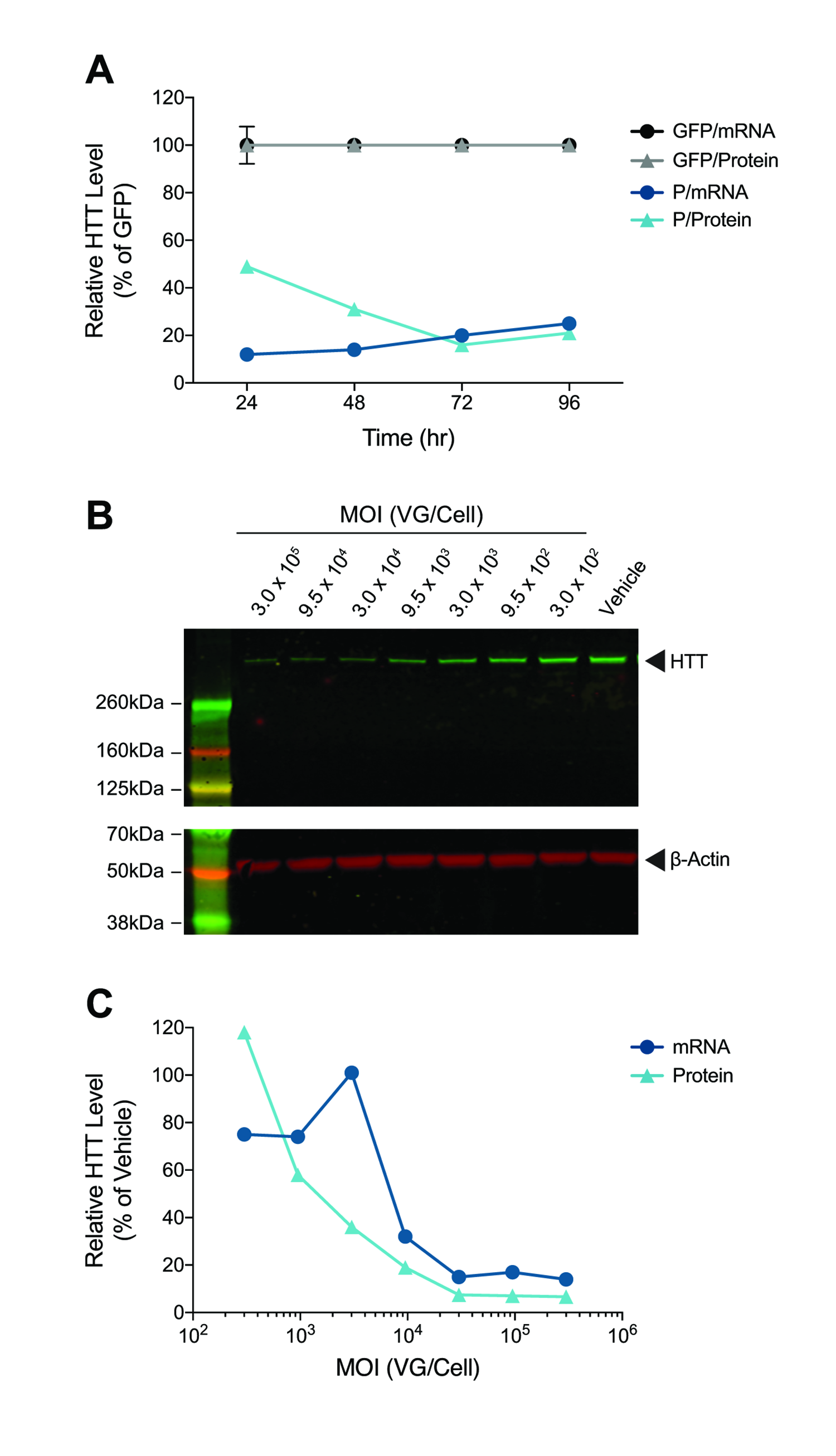


**Supplemental Figure S3.** Time- and dose-dependent HTT mRNA and protein lowering in HEK293T cells after transduction with treatment P. Relative HTT mRNA and protein levels were measured by RT-qPCR and Western blot, respectively, at (**A**) 24, 48, 72 or 96 hours following treatment P transduction at an MOI of 2 x 10^5^ VG/cell, and (**B, C**) 48 hours following treatment P transduction at an MOI of 0, 3.0 x 10^2^, 9.5 x 10^2^, 3.0 x 10^3^, 9.5 x 10^3^, 3.0 x 10^4^, 9.5 x 10^4^, or 3.0 x 10^5^ VG/cell. Controls comprised AAV.GFP for the time course experiment and vehicle for the dose-response experiment. Human HTT mRNA levels as well as mRNA levels of the endogenous reference gene XPNPEP1 were measured by RT-qPCR. Human HTT mRNA levels were normalized to XPNPEP1 mRNA levels, and then further normalized to the corresponding control treatment. HTT protein levels were normalized to the corresponding β-actin signal which served as a loading control for each sample and then further normalized to the corresponding control treatment. For relative HTT mRNA levels, the group mean ± standard deviation (*N*=2) is shown for each treatment. For relative HTT protein levels, the single determination is shown.
