# Supplementary material for: Efficient and Precise Processing of the Optimized Primary Artificial MicroRNA in a Huntingtin-Lowering Adeno-Associated Viral Gene Therapy In Vitro and in Mice and Nonhuman Primates
Source: Hum Gene Ther. 2022 Jan 17;33(1-2):37–60. doi: 10.1089/hum.2021.221 (PMC10112875; doi:10.1089/hum.2021.221)
Supplement: Supplemental data [file Suppl_FigureS4.docx]

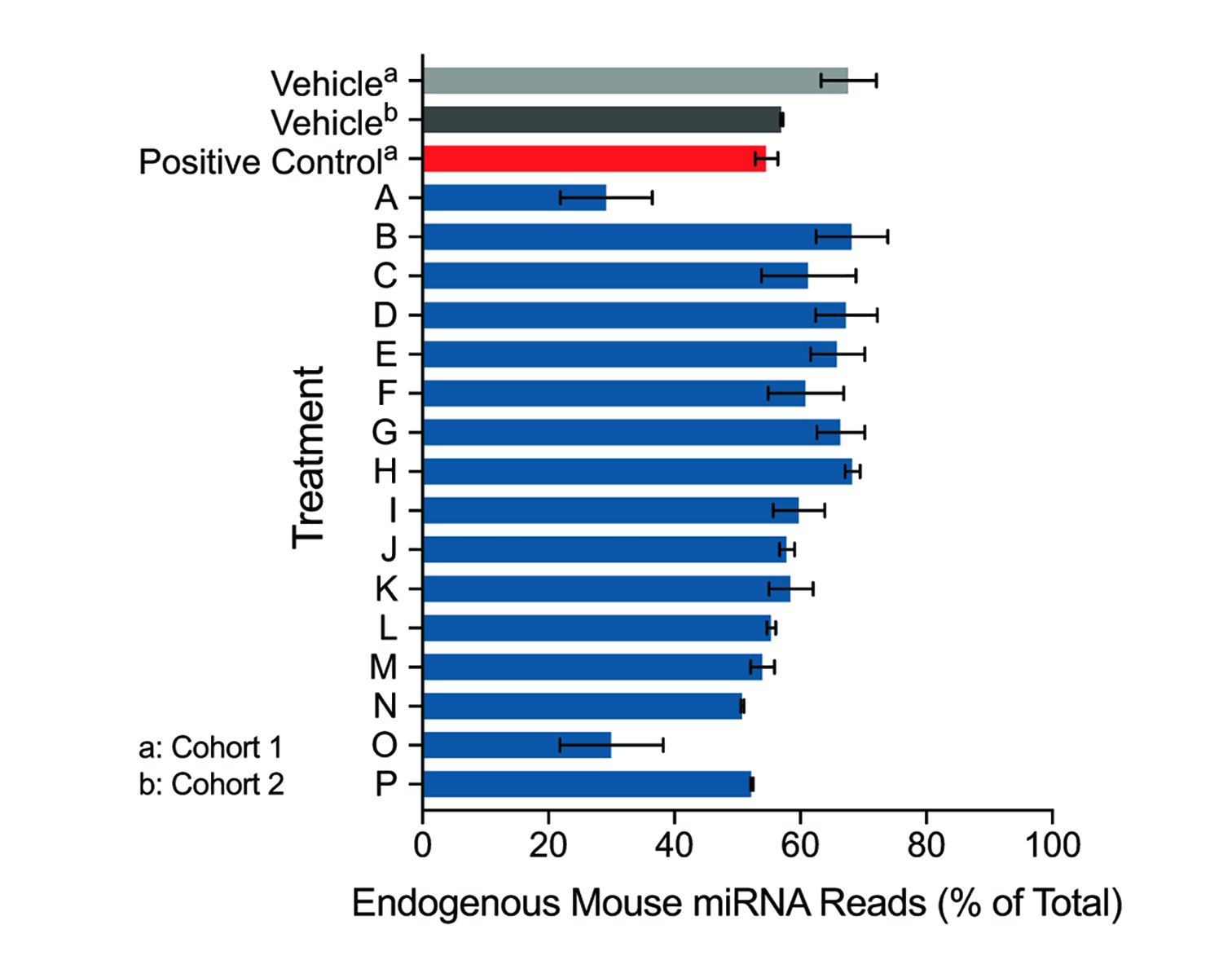


**Supplemental Figure S4.** Endogenous miRNA levels in YAC128 mouse striatum 4 weeks after bilateral intrastriatal injection of AAV1-packaged pri-amiRNAs targeting HTT at a dose of 2.6 x10^10^ to 6.0 x10^10^ VG per animal, administered to two cohorts of mice. Small RNA sequencing was carried out for sixteen pre-candidate pri-amiRNAs (cohort 1: A-H; cohort 2: I-P), positive control (cohort 1) and vehicle (cohorts 1 and 2). Endogenous miRNA levels are expressed as the percentage of total reads that aligned to annotated *Mus musculus* miRNA hairpin sequences. N=3 per group. The group mean ± standard deviation is shown for each treatment.
