# Supplementary material for: Efficient and Precise Processing of the Optimized Primary Artificial MicroRNA in a Huntingtin-Lowering Adeno-Associated Viral Gene Therapy In Vitro and in Mice and Nonhuman Primates
Source: Hum Gene Ther. 2022 Jan 17;33(1-2):37–60. doi: 10.1089/hum.2021.221 (PMC10112875; doi:10.1089/hum.2021.221)
Supplement: Supplemental data [file Suppl_FigureS5.docx]

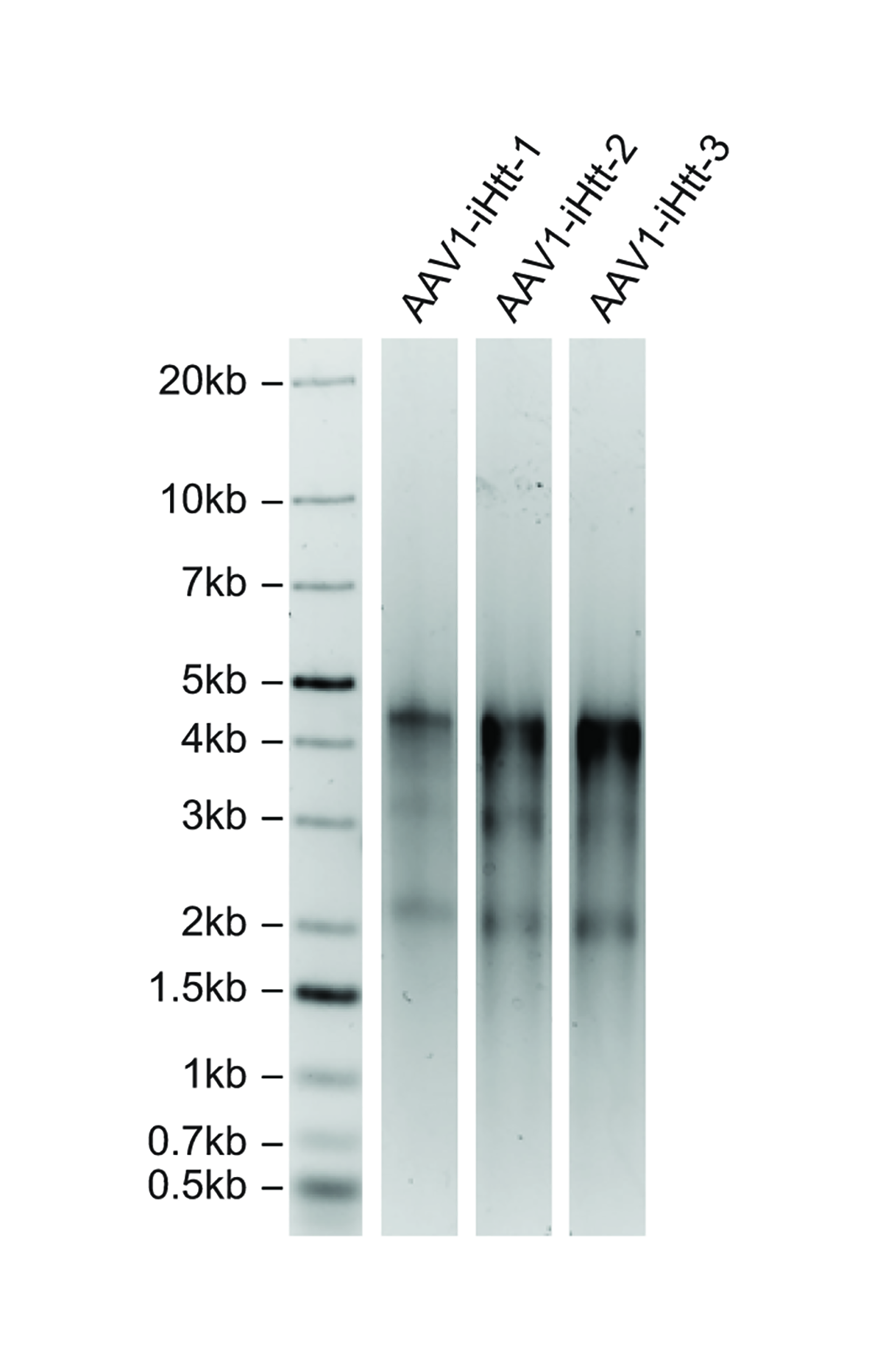


**Supplemental Figure S5.** Genome integrity for AAV1-pri-amiRNA batches evaluated in NHP. AAV1-iHtt-1, AAV1-iHtt-2 and AAV1-iHtt-3 (2.0 x10^10^ VG per lane) were run on a denaturing agarose gel and visualized with SYBR Gold. A Thermo GeneRuler 1kb Plus DNA ladder is shown in the far left lane for reference.
