# Supplementary material for: Efficient and Precise Processing of the Optimized Primary Artificial MicroRNA in a Huntingtin-Lowering Adeno-Associated Viral Gene Therapy In Vitro and in Mice and Nonhuman Primates
Source: Hum Gene Ther. 2022 Jan 17;33(1-2):37–60. doi: 10.1089/hum.2021.221 (PMC10112875; doi:10.1089/hum.2021.221)
Supplement: Supplemental data [file Suppl_FigureS6.docx]

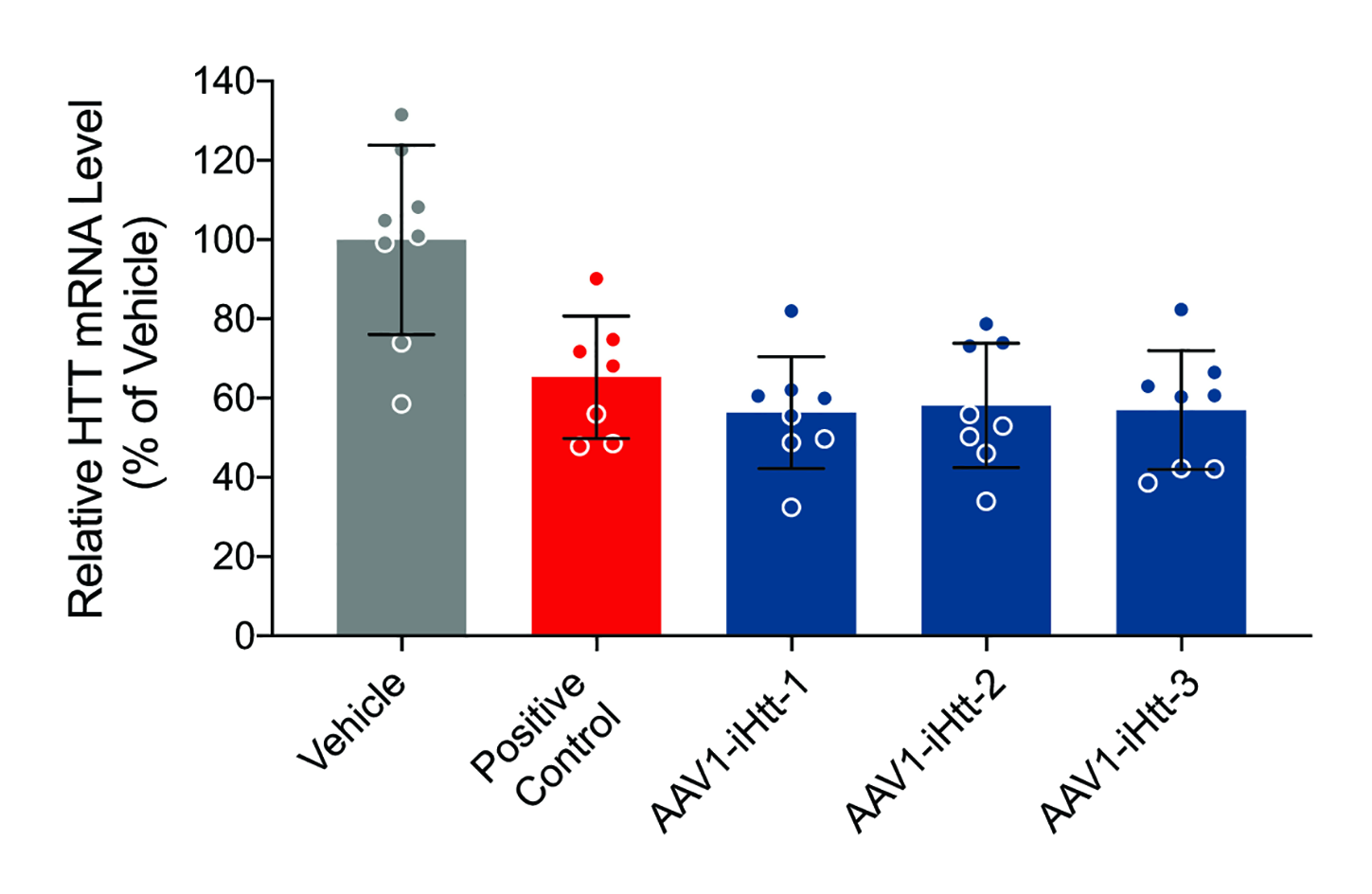


**Supplemental Figure S6.** Human HTT mRNA lowering in the YAC128 mouse striatum 4 weeks after bilateral intrastriatal injection of AAV1-iHtt-1, AAV1-iHtt-2, AAV1-iHtt-3 or a positive control at a dose of 3.6 x10^10^ VG per animal. Human mutant HTT mRNA levels as well as mRNA levels of the endogenous reference gene XPNPEP1 were measured by RT-qPCR. Human HTT mRNA levels were normalized to the mRNA level of XNPEP1, and then further normalized to the vehicle control group. Each symbol represents the average relative HTT mRNA level in the striatum of one animal. The group mean ± standard deviation is shown for each treatment. *N*=8 except for the positive control (*N*=7).
