# Supplementary material for: Efficient and Precise Processing of the Optimized Primary Artificial MicroRNA in a Huntingtin-Lowering Adeno-Associated Viral Gene Therapy In Vitro and in Mice and Nonhuman Primates
Source: Hum Gene Ther. 2022 Jan 17;33(1-2):37–60. doi: 10.1089/hum.2021.221 (PMC10112875; doi:10.1089/hum.2021.221)
Supplement: Supplemental data [file Suppl_FigureS7.docx]

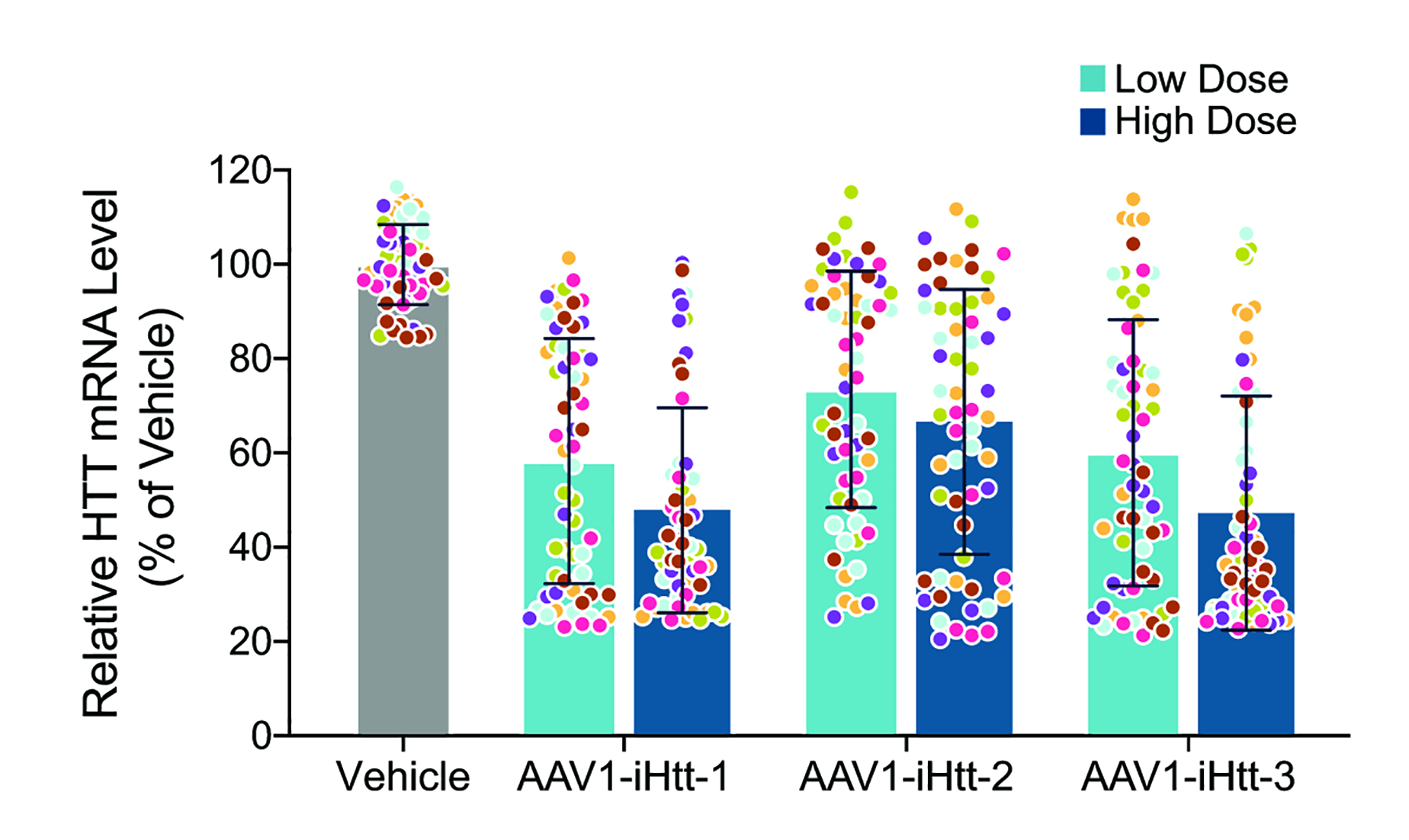


**Supplemental Figure S7.** HTT mRNA lowering in the NHP putamen 5 weeks after intraputaminal administration of low (9 × 10^10^ VG per putamen) or high (2.7 × 10^11^ VG per putamen) dose of AAV1-iHtt-1, AAV1-iHtt-2 or AAV1-iHtt-3. Tissue punches were measured by the bDNA assay for HTT, TBP, AARS and XPNPEP1 mRNA levels. HTT mRNA levels were normalized to the geometric mean of the mRNA levels of the three housekeeping genes TBP, AARS and XPNPEP1, and subsequently compared to the average normalized HTT mRNA level of the vehicle group. Each symbol represents the average relative HTT mRNA level in one putamen punch and each color within a group represents one hemisphere of one animal. The group mean ± standard deviation is shown for each treatment. *N*=60 tissue punches per AAV treatment per dose; *N*=60 tissue punches for vehicle.
