# Supplementary material for: Efficient and Precise Processing of the Optimized Primary Artificial MicroRNA in a Huntingtin-Lowering Adeno-Associated Viral Gene Therapy In Vitro and in Mice and Nonhuman Primates
Source: Hum Gene Ther. 2022 Jan 17;33(1-2):37–60. doi: 10.1089/hum.2021.221 (PMC10112875; doi:10.1089/hum.2021.221)
Supplement: Supplemental data [file Suppl_FigureS8.docx]

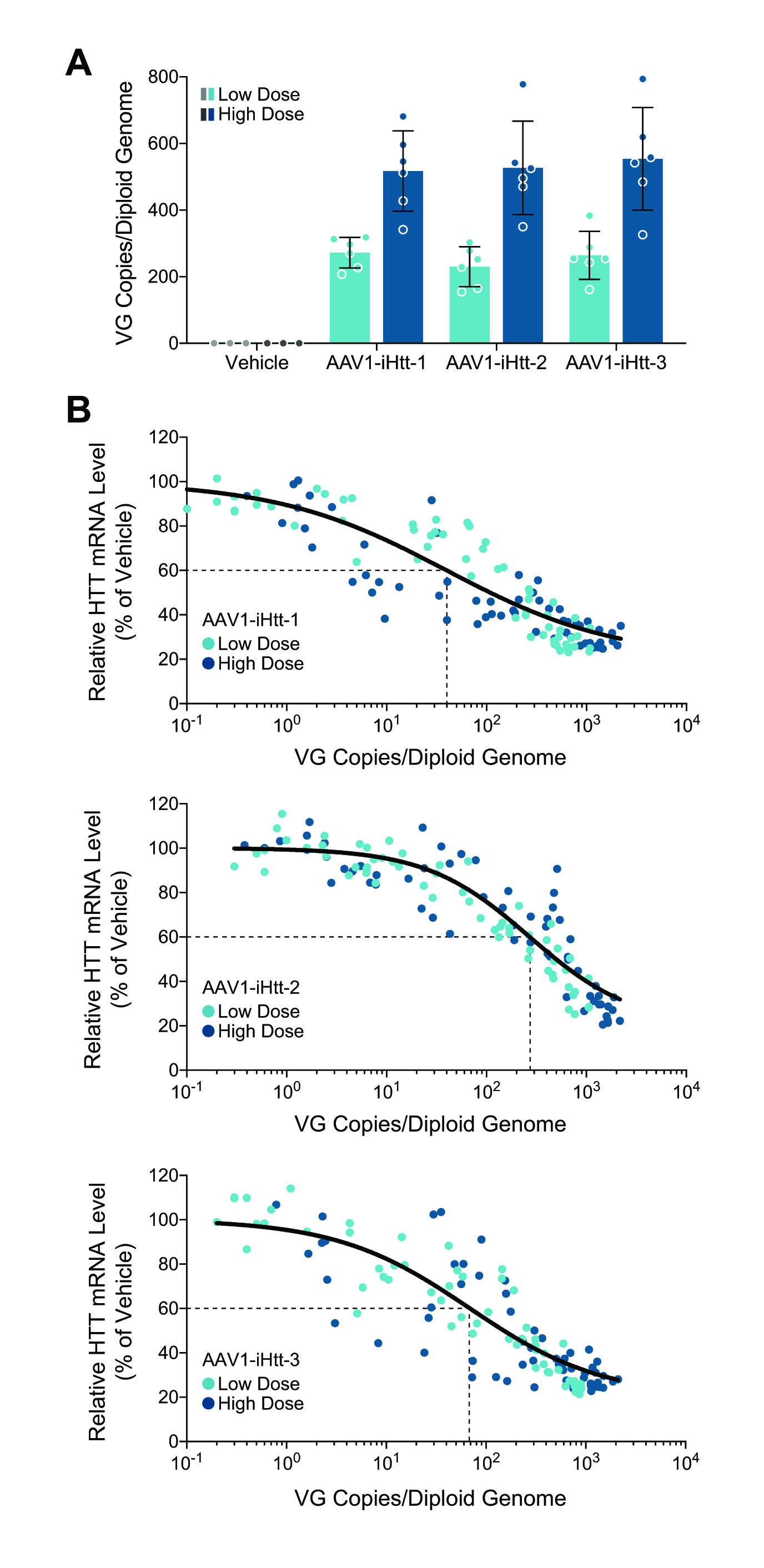


**Supplemental Figure S8.** Dose-dependent vector genome levels in NHP putamen 5 weeks after intraputaminal administration of low (9 × 10^10^ VG per putamen) or high (2.7 × 10^11^ VG per putamen) dose of AAV1-iHtt-1, AAV1-iHtt-2 or AAV1-iHtt-3. (**A**) Vector genome levels in the same tissue punches used for HTT mRNA measurements. Each symbol represents the average number of vector genome (VG) copies per diploid genome in the ten punches from one putamen. The group mean ± standard deviation is shown for each treatment. *N*=6 for AAV treatments, *N*=3 for vehicle. (**B**) Relative HTT mRNA level plotted as a function of vector genome level in the putamen. Top panel: AAV1-iHtt-1; middle panel: AAV1-iHtt-2; lower panel: AAV1-iHtt-3. Each symbol represents one putamen punch, with light blue circles for the low dose animals (*N*=60 punches) and dark blue circles for the high dose animals (*N*=60 punches). Each curve represents a nonlinear regression with a four-parameter fit, with the maximum and minimum asymptotes constrained at 100% and 20%, respectively. The dashed line indicates a relative HTT mRNA level midway between the two asymptotes, i.e., 60%.
